# Non-coronary peripheral arterial complications in people with type 2 diabetes: a Swedish retrospective cohort study

**DOI:** 10.1016/j.lanepe.2024.100888

**Published:** 2024-03-19

**Authors:** Araz Rawshani, Björn Eliasson, Jan Boren, Naveed Sattar, Deepak Bhatt, Linn El-Khalili, Joakim Nordanstig, Tarik Avdic, Joshua A. Beckman, Hertzel C. Gerstein, Darren K. McGuire, Elmir Omerovic, Aidin Rawshani

**Affiliations:** a Department of Molecular and Clinical Medicine, Institute of Medicine, University of Gothenburg, Sweden; b Wallenberg Laboratory for Cardiovascular and Metabolic Research, Institute of Medicine, University of Gothenburg, Sweden; c Dept of Medicine, Sahlgrenska University Hospital, Sweden; d Institute of Cardiovascular and Medical Sciences, British Heart Foundation Glasgow Cardiovascular Research Centre, Sweden; e Mount Sinai Fuster Heart Hospital, Icahn School of Medicine at Mount Sinai, New York, NY, USA; f Department of Vascular Surgery at the Sahlgrenska University Hospital, Gothenburg, Sweden; g Division of Cardiovascular Medicine, Department of Medicine, Vanderbilt University Medical Center, Nashville, TN, USA; h Population Health Research Institute, McMaster University and Hamilton Health Sciences, Hamilton, ON, Canada; i Department of Internal Medicine, University of Texas Southwestern Medical Center, Dallas, TX, USA; j Parkland Health and Hospital System, Dallas, TX, USA; k The Lundberg Laboratory for Diabetes Research, Department of Molecular and Clinical Medicine, University of Gothenburg, Sweden

**Keywords:** Peripheral arterial complications, Lower extremity artery disease, Extracranial large artery disease, Aortic complications, Diabetic foot disease, Type 2 diabetes mellitus

## Abstract

**Background:**

Few studies have explored long-term trends and risk factors for peripheral arterial complications in type 2 diabetes compared to the general population. Our research focuses on identifying optimal risk factors, their significance, risk associated with multifactorial risk factor control, and trends for these complications in diabetic patients versus general controls.

**Methods:**

This study included persons with type 2 diabetes mellitus entered into the Swedish National Diabetes Register 2001–2019 and controls matched for age-, sex- and county of residence. Outcomes comprised of extracranial large artery disease, aortic aneurysm, aortic dissection, lower extremity arterial disease and diabetes foot disease. Standardized incidence rates and Cox regression were used for analyses.

**Findings:**

The study comprises 655,250 persons with type 2 diabetes mellitus; average age 64.2; 43.8% women. Among persons with type 2 diabetes mellitus, the incidence rates per 100,000 person years for each non-coronary peripheral arterial complication event changed between 2001 and 2019 as follows: extracranial large artery disease 170.0–84.9; aortic aneurysm 40.6–69.2; aortic dissection 9.3 to 5.6; lower extremity artery disease from 338.8 to 190.8; and diabetic foot disease from 309.8 to 226.8. Baseline hemoglobin A1c (HbA1c), systolic blood pressure (SBP), smoking status and lipid levels were independently associated with all outcomes in the type 2 diabetes mellitus cohort. Within the cohort with type 2 diabetes mellitus, the risk for extracranial large artery disease and lower extremity artery disease increased in a stepwise fashion for each risk factor not within target. Excess risk for non-coronary peripheral arterial complications in the entire cohort for persons with type 2 diabetes mellitus, compared to matched controls, were as follows: extracranial large artery disease adjusted hazard ratio (HR) 1.69 (95% confidence interval (CI), 1.65–1.73), aortic aneurysm HR 0.89 (95% CI, 0.87–0.92), aortic dissection HR 0.51 (95% CI, 0.46–0.57) and lower extremity artery disease HR 2.59 (95% CI, 2.55–2.64).

**Interpretation:**

The incidence of non-coronary peripheral arterial complications has declined significantly among persons with type 2 diabetes mellitus, with the exception of aortic aneurysm. HbA1c, smoking and blood pressure demonstrated greatest relative contribution for outcomes and lower levels of cardiometabolic risk factors are associated with reduced relative risk of outcomes.

**Funding:**

Swedish Governmental and the County support of research and education of doctors, the 10.13039/501100003793Swedish Heart-Lung Foundation and Åke-Wibergs grant.

**Research in context**

**Evidence before this study**

Prior studies have demonstrated that individuals with type 2 diabetes mellitus are at a heightened risk for non-coronary peripheral arterial complications. To investigate this further, we conducted a comprehensive search on PubMed from the inception of the database until Mars 1, 2023. Our search employed the terms “peripheral arterial disease AND carotid disease AND aortic dissection AND aortic aneurysm AND type 2 diabetes AND incidence rates OR risk”. For each search, the term “peripheral arterial disease” was substituted with one of the specific outcomes evaluated in our study. To deepen our understanding of this association, we utilized nationwide registry data to evaluate the incidence and risk across a spectrum of arterial diseases with over two decades of follow-up. There is limited evidence regarding the rates and risk associations between type 2 diabetes mellitus, cardiometabolic risk factors, optimal risk factor levels and their relative importance for the development of both atherosclerotic and non-atherosclerotic peripheral arterial complications within a well-defined cohort. While considerable research has illustrated the risk factors contributing to atherosclerotic lower extremity arterial disease, there remains a lack of clarity on the disparate effects of these factors on different arterial complications, especially in differentiating the risks between type 2 diabetes mellitus and other diabetes types.

**Added value of this study**

In a large observational study conducted between 2001 and 2019, we compared individuals with type 2 diabetes mellitus to matched controls from the general population. Our investigation leverages extensive registry data to illustrate the trends in non-coronary peripheral arterial complications among individuals with type 2 diabetes mellitus compared to the general population. We have discovered significant reductions in the rates of atherosclerotic lower extremity arterial disease and diabetic foot disease, contrasting with a rise in aortic aneurysms, particularly thoracic aortic aneurysms in the type 2 diabetes mellitus cohort. Notably, while type 2 diabetes mellitus heightens the risk for atherosclerotic non-coronary peripheral arterial complications, it appears to confer a lower risk for aortic diseases compared to controls. Our study also underscores the profound impact of smoking, blood pressure, and glycated hemoglobin (HbA1c) levels on peripheral arterial outcomes. Remarkably, better management of these risk factors correlates with a lower risk of non-coronary peripheral arterial complications, except for lower extremity artery disease, where type 2 diabetes mellitus retains a modest risk elevation. These interventions hold the greatest potential for reducing the risk associated with peripheral arterial complications.

**Implications of all the available evidence**

The therapeutic management of type 2 diabetes mellitus has traditionally centered on traditional atherosclerotic cardiovascular disease, but our findings suggest a shifting paradigm for non-coronary peripheral arterial complications, with diabetic foot disease becoming increasingly prevalent. The study emphasizes the differential impact of cardiometabolic risk factors on peripheral arterial complications in comparison to cardiovascular disease. Glycated hemoglobin levels emerge as a particularly influential factor for peripheral arterial conditions. Additionally, the rise in aortic aneurysms and the paradoxical association of higher HbA1c levels with a reduced risk of aortic complications hint at a distinct pathophysiological process in the aortic disease of people with type 2 diabetes mellitus. This underlines the need for a nuanced approach to risk factor management, tailored to the specific arterial condition being addressed.

## Introduction

Type 2 diabetes mellitus is a complex cardiometabolic condition that has well-established consequences for cardiovascular disease risk.^1^, ^2^, ^3^ Results from epidemiological studies have demonstrated that individuals both with and without type 2 diabetes mellitus have experienced a decrease in the incidence and risk of lower extremity artery disease.^4^, ^5^, ^6^, ^7^, ^8^ These findings suggest a need to investigate the relative risk increment for lower extremity artery disease associated with type 2 diabetes mellitus in a contemporary cohort. Changes in incidence and prevalence of different manifestations of cardiovascular disease may signify a shift in cardiovascular complications and/or a changing risk factor milieu for disease development and few studies have investigated trends and risk associations across the whole peripheral arterial tree. Previous research has demonstrated that diabetes is strongly linked to both lower extremity artery disease and diabetic foot disease, with lower extremity artery disease and the progression of diabetes foot disease being interrelated.^9^, ^10^, ^11^ Conversely, there is a negative association between diabetes mellitus and aortic aneurysm.^12^, ^13^, ^14^ The present study aims to investigate a) recent trends in incidence for peripheral arterial complications among persons with and without type 2 diabetes mellitus; b) comparative risk between persons with type 2 diabetes mellitus versus matched controls, and c) among persons with type 2 diabetes mellitus, the relative prognostic importance of various risk markers for non-coronary peripheral arterial complications using data from the Swedish National Diabetes Registry and other national health registries.

## Methods

### Study design and support

This nationwide observational study was approved by the Ethical Review Authority (2020-04796) and all individuals with diabetes provided written informed consent for participation prior to inclusion into the registry. The authors had full access to the complete data in the study and take responsibility for the integrity of the data and data analysis. Information for matched controls was retrieved from the government agency Statistics Sweden (SCB). Because of the sensitive nature of the data collected for this study, access to the datasets is available from the sources stated in the paper on request to the data providers, fulfilling the legal and regulatory requirements, and with approval from the Swedish Ethical Review Authority.

### Data sources and study cohort

The study uses data from the Swedish National Diabetes Registry (NDR) and includes information on risk factors, complications of diabetes and medications for patients 18 years of age or older.^3^^,^^15^, ^16^, ^17^ The NDR includes >90% of all individuals in Sweden with type 2 diabetes mellitus and the diagnosis was defined using an epidemiological definition that treatment with diet with or without the use of oral antihyperglycemic agents or treatment with insulin with or without the use of oral antihyperglycemic agents; the latter category only applied to patients who were 40 years of age or older at the time of diabetes diagnosis. Furthermore, for those with missing information on diabetes classification according to the epidemiological definition, diabetes status was determined through a clinical assessment conducted by physicians and were identified as people with type 2 diabetes mellitus. The epidemiological definition has high validity and the concordance between the epidemiological definition and the physician’s classification of diabetes type is roughly 95%. In conjunction with the initial registration of each participant with type 2 diabetes mellitus into the NDR, five control individuals without diabetes diagnosis who were matched for age, sex, and county of residence, were randomly selected for each registry participant with type 2 diabetes mellitus. Data on matched controls were retrieved from the SCB. The type 2 diabetes mellitus study population was composed of persons who had at least one observation documented in the registry between January 1st, 2001, and December 31st, 2019. Persons with type 2 diabetes mellitus who met any of the pre-defined exclusion criteria, i.e., any form of peripheral arterial complications at baseline, were excluded along with all their matched controls. However, when matched controls met any exclusion criteria, they were excluded separately, i.e., without their matched persons with type 2 diabetes mellitus. This resulted in a matching ratio of 1:4.8 for persons with type 2 diabetes mellitus and matched controls.

### Outcomes

Information on outcomes that were assessed were retrieved from the Swedish in- and outpatient registry. The following incident diagnoses and outcomes were analyzed: extracranial large artery disease (includes atherosclerotic disease of all extracranial cerebrovascular arteries such as carotid- and vertebral arteries), thoracic, thoracoabdominal or abdominal aortic aneurysm, Stanford type A and B aortic dissection, lower extremity arterial disease (atherosclerotic narrowing or blockage of larger arteries from the infrarenal aorta down to the foot) and diabetic foot disease, which includes ICD-codes for diabetic foot syndrome-related microangiopathic- or neuropathic complications such as ulcers, infections and potential for or development of gangrene. For each aortic complication, both thoracic-, thoracoabdominal and abdominal outcomes were included. The incident diagnoses and outcomes were identified using the International Classification of Disease (ICD) version 10 coding. The specific ICD-codes included for each outcome measure are listed in Supplementary Table S1. Individuals were followed until December 31, 2019, or until an event or death occurred. The positive predictive value is high for outcome diagnoses.^18^

### Statistical analyses

Regression models include a range of variables, such as, age, sex, socioeconomic factors (i.e., education level, income, civil status, and ethnicity), pharmacological medications (i.e., cardiometabolic-, anticoagulant- and antithrombotic drugs), comorbidities (cerebrovascular disease, heart failure, hypertension, coronary artery disease, chronic obstructive pulmonary disease, dementia, end-stage renal disease and cancer), and risk factors. The cardiometabolic risk factors comprise of glycated hemoglobin (HbA1c), systolic (SBP) and diastolic blood pressure (DBP), low density lipoprotein cholesterol (LDL-C), high density lipoprotein cholesterol (HDL-C), total cholesterol, triglycerides, duration of diabetes, body mass index, estimated glomerular filtration rate (eGFR), and smoking status. Information of risk factors for matched controls were not available. Further details on the regression modeling can be found in the statistical methods section of the Supplementary appendix.

### Standardized incidence rates and cumulative survival probability after the onset of a peripheral arterial complication

**Fig. 1:**
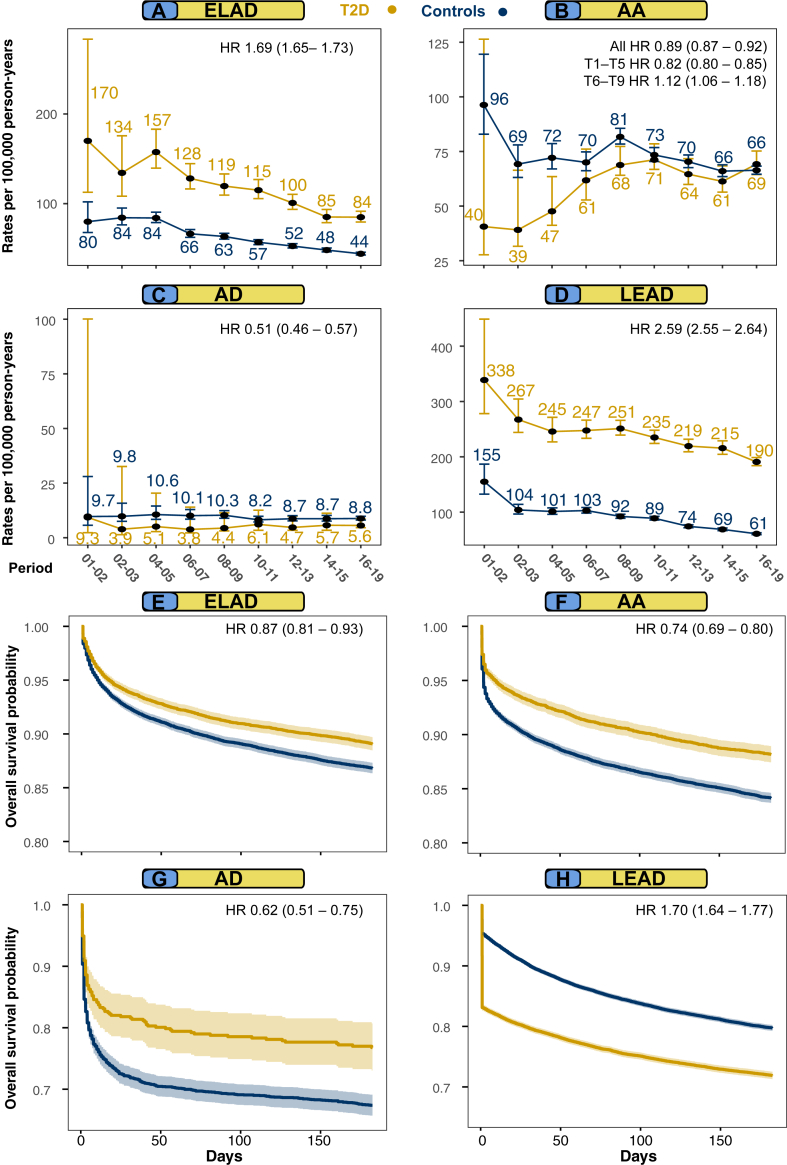
Standardized incidence rates for all outcomes and all-cause mortality after onset of peripheral arterial outcomes among persons with type 2 diabetes and matched controls. **Legend**: Age- and sex standardized incidence rates for all outcomes compared with controls from the general population (Panel A–D). Kaplan–Meier survival curves for all-cause mortality after the onset of different peripheral arterial outcomes (Panel E–H). Hazard ratios in Kaplan–Meier panels presents risk for patients with type 2 diabetes, adjusted for age and sex during 6 months of follow-up after onset of peripheral arterial complications. Hazard ratios in Panel A–D shows excess risk for diabetes in the entire cohort, adjusting for age, sex and category (i.e., either diabetic or control), except for the aortic aneurysm group where hazard ratios are presented for two distinct time periods.

The study interval from 2001 to 2019 was partitioned into two-year segments, with the exception of the last three-year period. Direct standardization was used to ensure the comparability of incidence rates across the age and sex distributions of the initial time period. The study cohort was classified according to five age categories: <45, 45–54, 55–64, 65–74, and ≥ 75 years of age. Participants that die during the follow-up or withdraw from the NDR, do not contribute person-years or events to the subsequent time periods. It is noteworthy that the incidence of participants leaving the registry is exceedingly rare, if not negligible. Each Kaplan–Meier survival curve include a subset of the cohort comprising study participants who had experienced a peripheral arterial complication during follow-up. The survival time, in this context, is determined by subtracting the time of death (either due to all-cause mortality are at the end of the follow-up) from the time of survival calculated from the entry of each participant into the registry until the occurrence of a peripheral arterial complication. To illustrate, for lower extremity artery disease (Fig. 1 Panel H), we included all individuals who experienced this event during the follow-up. The survival time for lower extremity artery disease was defined as the duration in days from their inclusion in the registry until the onset of lower extremity artery disease. Subsequently, we utilized the survival time from the date of experiencing lower extremity artery disease until the point of death or the conclusion of the follow-up period. Supplementary Fig. S2 shows Kaplan–Meier survival curves for peripheral arterial complications for all outcomes from 6 months and onwards, since changes in all-cause mortality stabilizes after the initial 6 months.

### Association between risk factors and peripheral arterial complications

The study employed Cox proportional hazards models to assess the association between specific cardiometabolic risk factors and outcomes. As data regarding cardiometabolic risk factors were not available for the control group, these analyses were conducted solely on the type 2 diabetes mellitus cohort. The regression models are intended to display risk association in comparison to therapeutic target levels. To detect non-linear associations, the Cox regression models were fitted with restricted cubic splines with three evenly spaced knots. Initially, the model included five knots for continuous predictors, but there was no improvement in performance beyond using three knots spaced evenly. The cardiometabolic risk factors that were evaluated in these models included glycated hemoglobin (HbA1c, mmol/mol), systolic blood pressure (SBP, mmHg), diastolic blood pressure (DBP, mmHg), body mass index (BMI, kg/m^2^), estimated glomerular filtration rate (eGFR, ml/min/1.73 m^2^), low-density lipoprotein cholesterol (LDL-C, mg/dL), and triglycerides (TG, mg/dL).

The models were adjusted for the following covariates: age, sex, physical activity (0–5), ethnicity, marital status, income level, educational level, comorbidities, and pharmacological treatment for cardiovascular conditions. The risk factors values associated with the lowest risk for each outcome was estimated using these models. An example of the Cox regression modeling strategy, estimating the optimal levels for glycated hemoglobin, is presented in the Supplementary material.

### Multifactorial risk factor control

Cox regression models were used to examine the possibility of reducing excess risk of non-coronary peripheral arterial complications by assessing the relationship between the co-occurrence of multiple risk factors and the likelihood of developing outcomes. Participants with type 2 diabetes mellitus were assigned to various groups according to the number of risk factors that did not fall within evidence-based target levels, at baseline. The risk factors were modeled as categorical variables (the risk factors within the target ranges: yes/no). The risk factors included in these models (along with their corresponding cut-off values) were glycated hemoglobin (HbA1c; ≥7.0%), blood pressure (systolic blood pressure [SBP; ≥130 mmHg] and diastolic blood pressure [DBP; ≥80 mmHg]), current smoking, low-density lipoprotein cholesterol (LDL-C; ≥97 mg/dL), and the presence of micro- or macroalbuminuria.

The Cox models were modified to account for sex, age, baseline comorbidities, socioeconomic variables, treatment with either antihypertensives, statins, antithrombotic or anticoagulant medications. Information of pharmacological treatment was retrieved from the Swedish prescribed drug registry. Additionally, the models were adjusted to account for the duration of type 2 diabetes by assigning matched controls to a duration of zero years whereas persons with type 2 diabetes mellitus had their duration of type 2 diabetes mellitus centralized around the pooled mean.

### Relative importance, excess- and competing risk

**Fig. 2:**
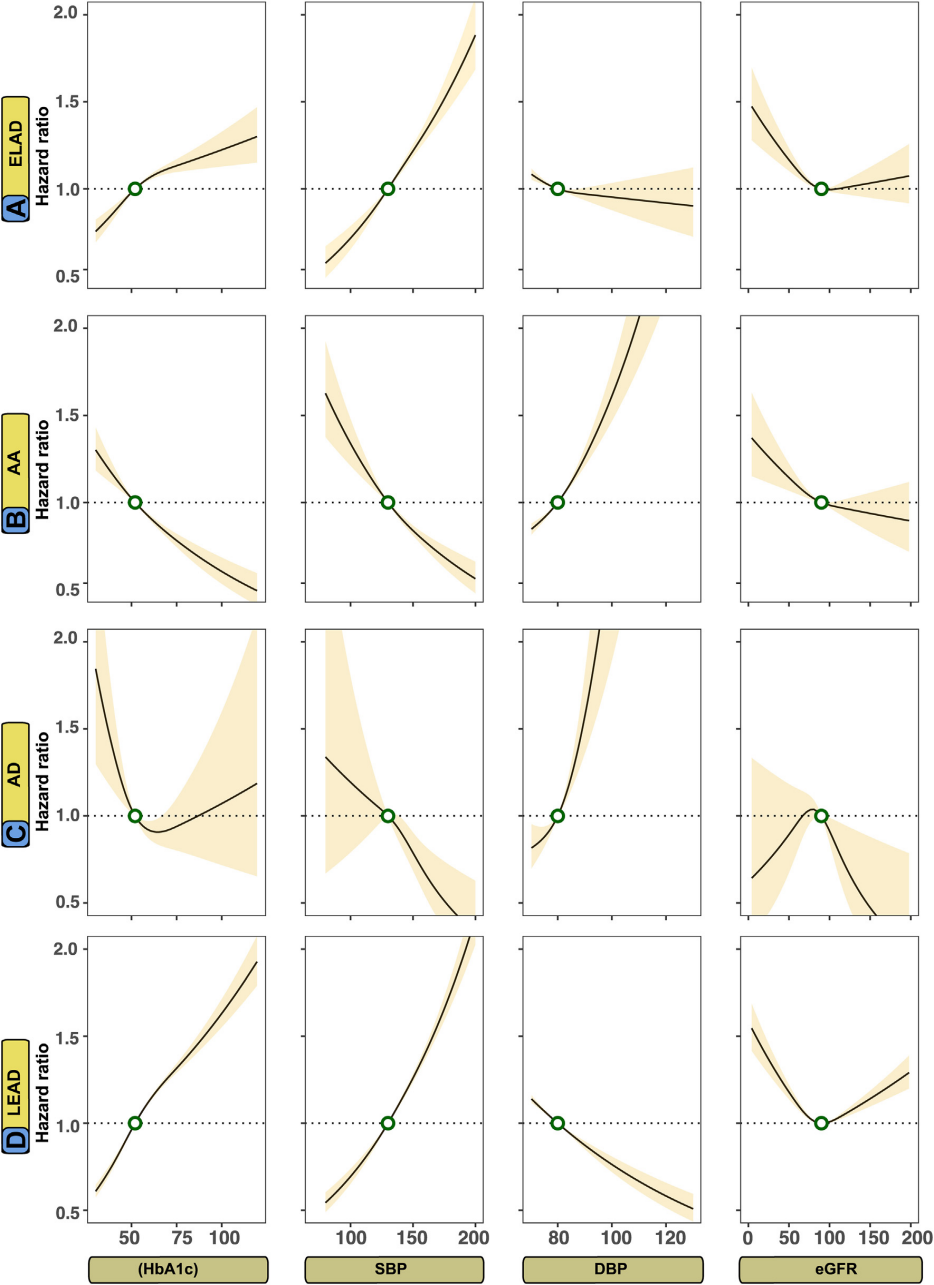
Adjusted risk association between levels of glycated hemoglobin, systolic blood pressure, diastolic blood pressure, and estimated glomerular filtration rate and peripheral arterial outcomes in persons with type 2 diabetes mellitus. **Legend**: A Cox model was constructed for each outcome and applied a prediction function to assess the relationship between selected risk factors and outcomes (Panel A–D). The dark lines indicate the hazard function and the shaded areas 95% confidence intervals. Continuous variables were modeled with restricted cubic splines. The following cut-off levels were used for risk factors: glycated hemoglobin (≥52 mmol/mol, SBP (≥130 mmHg), DBP (≥80 mmHg), and eGFR (≤90 ml/min/1.73 m^2^).

**Fig. 3:**
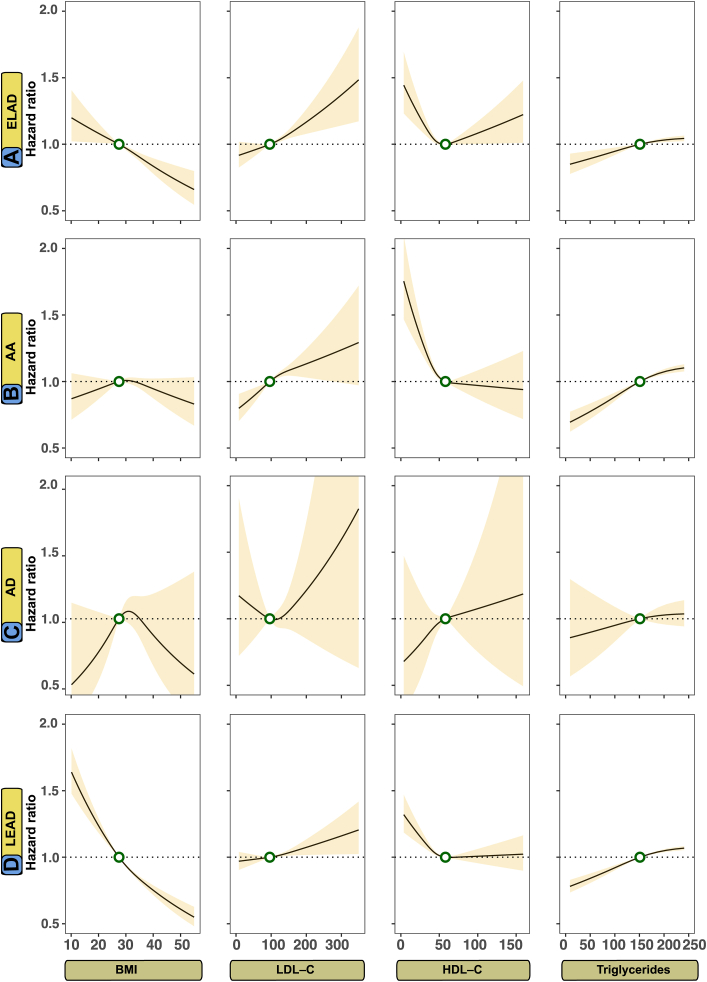
Adjusted risk association between levels of body mass index, low-density lipoprotein cholesterol, high-density lipoprotein cholesterol, and triglycerides for peripheral arterial outcomes in persons with type 2 diabetes mellitus. **Legend**: A Cox model was constructed for each outcome and applied a prediction function to assess the relationship between selected risk factors and outcomes (Panel A–D). The dark lines indicate the hazard function and the shaded areas 95% confidence intervals. Continuous variables were modeled with restricted cubic splines. The following cut-off levels were used for risk factors: BMI ≥ 27.5 kg/m^2^, LDL-C (≥96 mg/dL), HDL-C (≤60 mg/dL), and triglycerides (≥151 mg/dL).

A developed application for the Cox model was used to estimate the partial contribution of each predictor for peripheral arterial outcomes. The relative importance measure is an estimation of the predictive importance of each risk factor to the model. For the relative importance analyses, the explainable log-likelihood attributable to each risk factor was calculated, presented as the Wald χ^2^ (Chi-squared) statistic minus the degrees of freedom for each covariate. These regressions models were also adjusted for comorbidities, pharmacological treatment, sex and age. In Supplementary Fig. S3, hazard functions for aortic complications using competing risk regression models (Fine and Gray method) are presented for HbA1c, blood pressure variables and BMI, using similar model construction as Cox models in Figs. 2 and 3.

Proportional hazards assumption holds for all Cox regression models. The proportionality assumption was checked using Schoenfeld’s residual plots and the Grambsch–Therneau test, and we used stratification to incorporate variables which failed these checks. Missing data (ranging around 5–10%) were handled using multiple imputation by chained equations (MICE). Variables included in the imputation model are presented in Supplementary Table S2. A p-value of less than 0.05 was considered to indicate statistical significance. Calculations were performed using R version 4.3.1 (R Foundation for Statistical Computing) and analyses in RStudio.

### Role of the funding source

This study is supported by grants from the Swedish state under an agreement between the Swedish government and the county councils concerning economic support of research and education of doctors [ALFGBG-966187], the Swedish Heart and Lung Foundation [HLF 2019-0532] and Åke-Wibergs stiftelse [M22-0206]. The funding sponsors had no role in designing, analyzing, interpreting, writing or deciding to submit the paper for publication.

## Results

### Study population

In all, 655,250 persons with type 2 diabetes mellitus and 2,512,165 matched controls were included in the study. For those with type 2 diabetes mellitus, the mean age was 64.2 (SD 12.57) (Table 1). Cardiovascular disease and heart failure at baseline were roughly 2–3 times as frequent in participants with type 2 diabetes mellitus compared with controls, and they were also more often treated with anticoagulants, antithrombotic medications, statins, and antihypertensive medications. Median follow-up for people with type 2 diabetes mellitus in the entire cohort was 7.18 years. See Supplementary Fig. S1 for flowchart with information on study individuals and specific analyses. In Supplementary Table S3, baseline characteristics for persons with type 2 diabetes mellitus according to time-period (i.e., inclusion in the registry), are presented.

**Table 1:** Baseline characteristics for patients with type 2 diabetes according to age-categories and matched controls.

	Controls	Diabetes	Diabetes: <45	Diabetes: 45–54	Diabetes: 55–64	Diabetes: 65–74	Diabetes: ≥75
n	2,512,165	655,250	43,767	88,484	202,794	179,078	141,127
Sex = female %	1,201,717 (47.8)	286,897 (43.8)	18,478 (42.2)	33,139 (37.5)	81,114 (40.0)	77,837 (43.5)	76,329 (54.1)
Age (mean (SD))	62.27 (12.81)	64.24 (12.57)	37.59 (6.15)	50.13 (2.81)	60.18 (2.61)	69.30 (2.85)	80.80 (4.66)
Age-category (%)							
<45	220,767 (8.8)	43,767 (6.7)	43,767 (100.0)				
45–54	410,993 (16.4)	88,484 (13.5)		88,484 (100.0)			
55–64	832,015 (33.1)	202,794 (30.9)			202,794 (100.0)		
65–74	610,357 (24.3)	179,078 (27.3)				179,078 (100.0)	
≥75	438,033 (17.4)	141,127 (21.5)					141,127 (100.0)
Education n (%)							
Pre-secondary education ≤ 9 years	882,208 (35.1)	254,151 (38.8)	12,108 (27.7)	22,333 (25.2)	65,518 (32.3)	74,943 (41.8)	79,249 (56.2)
Secondary education > 9–12 years	979,399 (39.0)	286,575 (43.7)	22,177 (50.7)	46,812 (52.9)	96,120 (47.4)	74,368 (41.5)	47,098 (33.4)
Post-secondary education ≥ 12 years	650,558 (25.9)	114,524 (17.5)	9482 (21.7)	19,339 (21.9)	41,156 (20.3)	29,767 (16.6)	14,780 (10.5)
Civil status: married n (%)	1,257,346 (50.1)	337,629 (51.5)	17,707 (40.5)	43,382 (49.0)	111,146 (54.8)	103,067 (57.6)	62,327 (44.2)
Ethnicity = Scandinavian, n (%)	2,247,282 (89.5)	550,266 (84.0)	28,305 (64.7)	64,582 (73.0)	164,630 (81.2)	161,429 (90.1)	131,320 (93.1)
Income family quartiles n (%)							
Quartile 1	588,597 (23.4)	196,089 (29.9)	12,198 (27.9)	19,761 (22.3)	36,333 (17.9)	55,156 (30.8)	72,641 (51.5)
Quartile 2	607,603 (24.2)	185,433 (28.3)	12,536 (28.6)	22,431 (25.4)	43,501 (21.5)	59,174 (33.0)	47,791 (33.9)
Quartile 3	635,961 (25.3)	157,399 (24.0)	9832 (22.5)	20,030 (22.6)	78,372 (38.6)	35,829 (20.0)	13,336 (9.4)
Quartile 4	680,003 (27.1)	116,329 (17.8)	9201 (21.0)	26,262 (29.7)	44,588 (22.0)	28,919 (16.1)	7359 (5.2)
Income quartiles n (%)							
Quartile 1	552,011 (22.0)	150,457 (23.0)	10,505 (24.0)	14,748 (16.7)	37,579 (18.5)	40,858 (22.8)	46,767 (33.1)
Quartile 2	596,556 (23.7)	178,963 (27.3)	8197 (18.7)	15,884 (18.0)	43,510 (21.5)	55,688 (31.1)	55,684 (39.5)
Quartile 3	652,356 (26.0)	159,673 (24.4)	11,813 (27.0)	23,917 (27.0)	52,203 (25.7)	44,094 (24.6)	27,646 (19.6)
Quartile 4	711,241 (28.3)	166,157 (25.4)	13,252 (30.3)	33,935 (38.4)	69,502 (34.3)	38,438 (21.5)	11,030 (7.8)
Hypertension n (%)	313,949 (12.5)	185,227 (28.3)	3575 (8.2)	15,081 (17.0)	52,331 (25.8)	58,824 (32.8)	55,416 (39.3)
Heart failure n (%)	65,845 (2.6)	39,559 (6.0)	320 (0.7)	1621 (1.8)	7332 (3.6)	11,086 (6.2)	19,200 (13.6)
Coronary heart disease n (%)	161,359 (6.4)	96,843 (14.8)	653 (1.5)	5449 (6.2)	24,277 (12.0)	31,792 (17.8)	34,672 (24.6)
Chronic obstructive pulmonary disease n (%)	48,367 (1.9)	18,342 (2.8)	106 (0.2)	869 (1.0)	4624 (2.3)	6609 (3.7)	6134 (4.3)
Dementia n (%)	31,038 (1.2)	4857 (0.7)	12 (0.0)	47 (0.1)	464 (0.2)	977 (0.5)	3357 (2.4)
End-stage kidney disease n (%)	22,539 (0.9)	14,370 (2.2)	547 (1.2)	1271 (1.4)	3593 (1.8)	4009 (2.2)	4950 (3.5)
Cancer n (%)	262,144 (10.4)	71,322 (10.9)	672 (1.5)	2914 (3.3)	15,802 (7.8)	24,398 (13.6)	27,536 (19.5)
Antihypertensive medication n (%)	886,931 (35.3)	386,950 (59.1)	19,642 (44.9)	58,416 (66.0)	141,874 (70.0)	115,449 (64.5)	51,569 (36.5)
Statins n (%)	415,831 (16.6)	324,304 (49.5)	20,235 (46.2)	54,652 (61.8)	123,505 (60.9)	94,326 (52.7)	31,586 (22.4)
Anticoagulant medication n (%)	212,286 (8.5)	79,074 (12.1)	1396 (3.2)	5675 (6.4)	23,761 (11.7)	29,700 (16.6)	18,542 (13.1)
Antithrombotic medication n (%)	289,556 (11.5)	148,582 (22.7)	3933 (9.0)	17,999 (20.3)	53,631 (26.4)	49,446 (27.6)	23,573 (16.7)
Age at onset of diabetes (mean (SD))			35.84 (7.55)	47.78 (5.23)	57.02 (8.09)	64.93 (6.70)	74.33 (9.08)
Duration of diabetes (mean (SD))			1.98 (3.63)	2.52 (4.29)	3.17 (5.13)	4.16 (6.16)	5.51 (7.42)
Glycated hemoglobin levels (mean (SD))^a^			59.02 (20.55)	58.09 (19.29)	55.85 (17.68)	53.41 (15.14)	52.83 (13.25)
Current smoking n (%)			11,290 (25.8)	22,026 (24.9)	39,457 (19.5)	22,660 (12.7)	8455 (6.0)
Albuminuria n (%)							
No albuminuria			36,885 (84.3)	73,262 (82.8)	166,122 (81.9)	143,477 (80.1)	106,092 (75.2)
Normal albuminuria			204 (0.5)	391 (0.4)	896 (0.4)	773 (0.4)	540 (0.4)
Microalbuminuria			4988 (11.4)	10,852 (12.3)	25,954 (12.8)	24,236 (13.5)	22,289 (15.8)
Macroalbuminuria			1690 (3.9)	3979 (4.5)	9822 (4.8)	10,592 (5.9)	12,206 (8.6)
eGFR (mean (SD))^b^			113.73 (39.49)	98.52 (24.27)	89.24 (26.15)	78.50 (20.16)	66.56 (20.39)
Retinopathy n (%)			6034 (13.8)	13,490 (15.2)	32,292 (15.9)	31,188 (17.4)	27,129 (19.2)
Systolic blood pressure (mean (SD))			128.57 (15.63)	133.83 (16.17)	137.22 (16.64)	140.02 (16.93)	141.77 (18.17)
Diastolic blood pressure (mean (SD))			80.92 (10.56)	82.12 (10.04)	80.76 (9.68)	78.58 (9.40)	75.89 (9.57)
Total cholesterol (mean (SD))			198.89 (46.92)	201.51 (46.55)	197.98 (44.95)	194.48 (43.20)	192.94 (42.71)
High-density lipoprotein cholesterol (mean (SD))			41.86 (14.11)	44.57 (14.73)	47.74 (15.45)	50.80 (16.28)	54.30 (17.68)
Triglycerides (mean (SD))			219.28 (193.99)	207.87 (168.85)	185.74 (134.27)	169.05 (108.29)	159.95 (101.20)
Low-density lipoprotein cholesterol (mean (SD))			117.63 (38.14)	119.01 (38.54)	115.98 (38.33)	112.02 (37.23)	108.73 (36.23)
S-creatinine (mean (SD))			64.94 (20.79)	70.18 (21.88)	74.48 (23.34)	80.32 (27.12)	89.31 (32.14)
Body mass index (mean (SD))			33.29 (7.02)	31.94 (6.08)	30.73 (5.48)	29.78 (5.06)	28.34 (4.66)
Physical activity n (%)							
1 = Never			6909 (15.8)	13,671 (15.5)	31,948 (15.8)	29,535 (16.5)	33,967 (24.1)
2 = <1 time/week			6204 (14.2)	12,469 (14.1)	26,934 (13.3)	22,140 (12.4)	19,571 (13.9)
3 = 1–2 times/week			8950 (20.4)	18,295 (20.7)	40,861 (20.1)	34,053 (19.0)	26,167 (18.5)
4 = 3–5 times/week			9994 (22.8)	19,673 (22.2)	44,537 (22.0)	38,243 (21.4)	25,514 (18.1)
Daily			11,710 (26.8)	24,376 (27.5)	58,514 (28.9)	55,107 (30.8)	35,908 (25.4)

Controls are individuals, matched for age, sex and county, who were randomly selected from the general population.

a Concentrations of glycated hemoglobin are based on values from the International Federation of Clinical Chemistry.

b Glomerular filtration rate was estimated using the Modification of Diet in Renal Disease Study Equation.

### Standardized incidence rates and all-cause mortality after onset of peripheral arterial complications

**Fig. 5:**
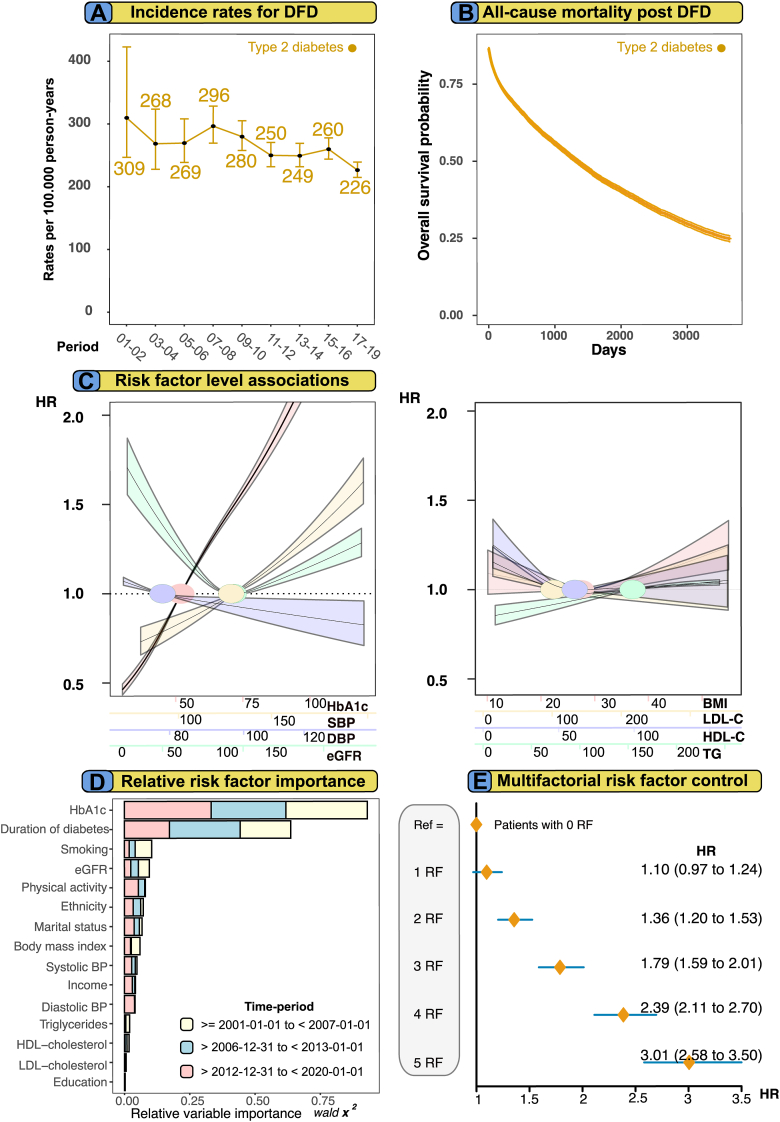
Analyses for diabetic foot disease in patients with type 2 diabetes mellitus. **Legend**: All principal analyses are performed for diabetic foot disease. Panel A–B shows incidence rates and Kaplan–Meier curves for patients with diabetes only. Panel C shows adjusted risk association for cardiometabolic risk factors and Panel D shows relative importance of risk factors for diabetic foot disease. Panel E shows excess risk for diabetic foot disease in patients with type 2 diabetes mellitus and no risk factors compared with patients that had 1-5 risk factors at baseline. Matched controls do not develop diabetic foot-related syndrome but other causes of smaller blood vessel dysfunction in foot.

The numbers of events during each period, as well as crude and standardized incidence rates are reported as the number of events per 100,000 person-years for extracranial large artery disease, lower extremity artery disease, diabetic foot disease, aortic aneurysm, and aortic dissection. Results for incidence rates are presented in Supplementary Table S4. Substantial reductions were observed over time regarding incidences for extracranial large artery disease and lower extremity artery disease, in both persons with type 2 diabetes mellitus and in matched controls and for diabetic foot disease in individuals with diabetes. During the study period, the incidence rates for extracranial large artery diosease decreased from 170.0 to 84.9 in the population with type 2 diabetes mellitus and from 79.9 to 44.1 in the controls (Fig. 1 Panel A). For aortic aneurysm, the rates increased from 40.6 to 69.2 in type 2 diabetes mellitus and decreased for controls (96.3–66.4) (Fig. 1 Panel B), whereas rates for aortic dissection changed from 9.3 to 5.6 for type 2 diabetes mellitus and 9.7 to 8.8 for controls. Rates for lower extremity artery disease changed from 338.8 to 190.8 in type 2 diabetes mellitus and from 155.0 to 60.9 in controls (Fig. 1 Panel D), whilst diabetic foot disease changed from 309.8 to 226.8 in type 2 diabetes mellitus (Fig. 5 Panel A). Fig. 1 Panel E–H shows Kaplan–Meier survival curves for all-cause mortality, during the first 150 days, after the onset of peripheral arterial outcomes in persons with type 2 diabetes mellitus and matched controls. Individuals with type 2 diabetes mellitus and the general population roughly had a 40–60% all-cause mortality rate within 3650 days (10.9 years) after the onset of any peripheral arterial outcomes, with the highest mortality rates in persons with lower extremity artery disease (Supplementary Fig. S2 Panel A–D). Patients with type 2 diabetes mellitus had substantially lower survival rates after onset of lower extremity artery disease, while controls had better survival rates for extracranial large artery disease and aortic disease, during the first 150 days (Fig. 1 Panel E–H).

The overall adjusted excess risk for persons with type 2 diabetes mellitus were as follows: extracranial large artery disease HR 1.65 (95% CI, 1.62–1.70), aortic aneurysm HR 0.89 (95% CI, 0.86–0.91), aortic dissection HR 0.52 (95% CI, 0.47–0.57), lower extremity artery disease HR 2.51 (95% CI, 2.46–2.55) (Supplementary Table S5).

### Optimal levels for cardiometabolic risk factors in peripheral arterial complications

Figs. 2 and 3 show the adjusted hazard functions between a series of cardiometabolic risk factors and non-coronary peripheral arterial outcomes in persons with type 2 diabetes mellitus. For atherosclerotic peripheral artery disease, elevated HbA1c, SBP and LDL-C levels were associated with higher risk and lower levels than contemporary target levels were associated with lower risks, whereas higher levels of DBP and BMI were associated with lower risk. Higher levels of DBP, LDL-C and TG increases the risk for aortic disease, whereas higher SBP and HbA1c seems protective of aortic disease.

### Modifiable risk factors beyond target and relative importance for outcomes

**Fig. 4:**
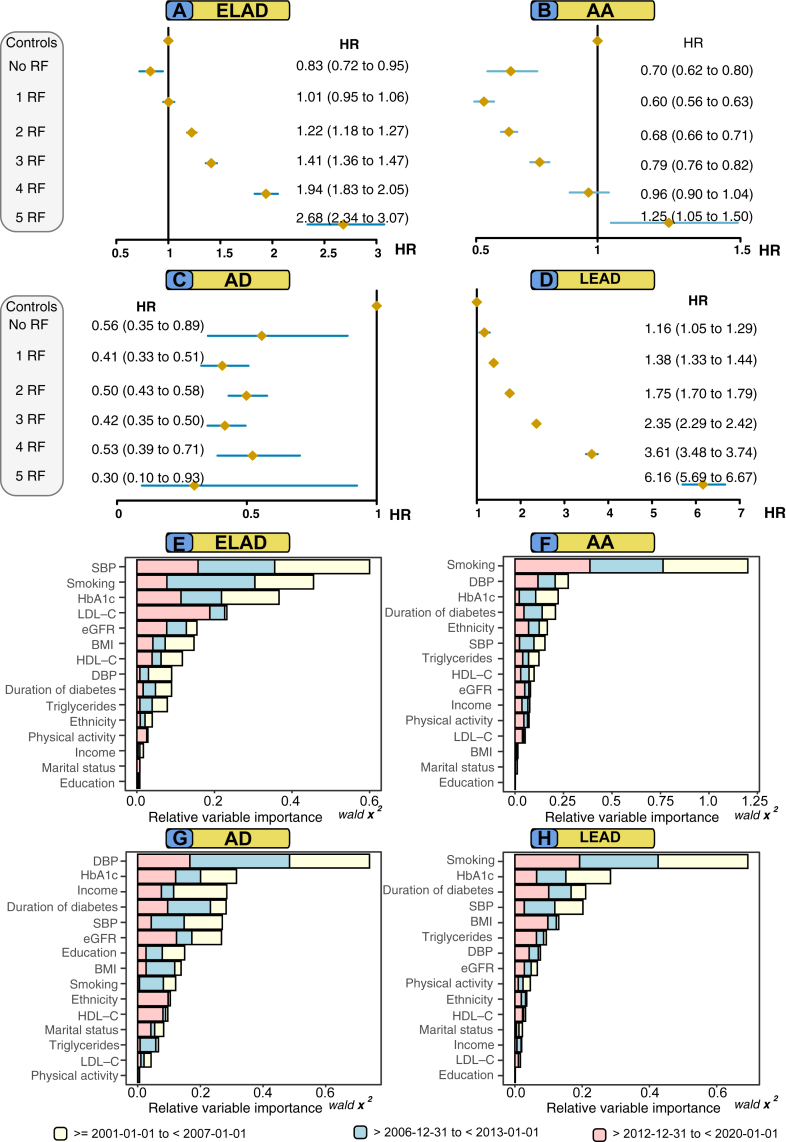
Adjusted hazard ratios for peripheral arterial outcomes, according to number of risk factor variables outside target range among persons type 2 diabetes mellitus, as compared with matched controls. Relative variable importance generated from the Cox proportional hazards models for outcomes, among persons with type 2 diabetes mellitus. **Legend**: Hazard ratios show the excess risk of each outcome among persons with type 2 diabetes mellitus, compared with matched controls from the general population, according to number of risk factors (scale, none to five) that were outside target ranges (Panel A–D). Relative variable importance of risk factors is displayed in Panel E–H. Variables with higher importance measures demonstrated a high predictive performance and are deemed important for modeling the outcome.

Fig. 4 Panel A–D, displays adjusted hazard ratios for peripheral arterial complications in six subgroups of the type 2 diabetes mellitus cohort, defined by levels beyond target of 5 modifiable risk factors (i.e., ranging from none to all within target). These risk factors included HbA1c, blood pressure (SBP and DBP), LDL-C, smoking status and presence of albuminuria. In those with type 2 diabetes mellitus, the risk of atherosclerotic peripheral arterial disease was incrementally higher for each additional risk factor not within the target range, whereas individuals with optimal risk factor control displayed virtually no excess risk. Hazard ratios for optimal risk factor control of extracranial large artery disease and lower extremity artery disease was 0.83 (0.72–0.95) and 1.16 (1.05–1.29), respectively.

Persons with all risk factors outside target range (5 RF) displayed the highest HR for extracranial large artery disease and lower extremity artery disease, 2.68 (95% CI, 2.34–3.07) and 6.16 (95% CI, 5.69–6.67), respectively (Fig. 4 Panel A & Panel D). Adjusted hazard ratio for aortic disease revealed lower hazard ratios in persons with type 2 diabetes mellitus, across all risk factor groups for aortic disease, with the exception of those with 5 RF and aortic aneurysm (HR 1.25, 95% CI 1.05–1.50) (Fig. 4 Panel B). The pattern for aortic dissection was different and no incremental risk increase for people with type 2 diabetes mellitus was observed (Fig. 4 Panel C). Individuals with type 2 diabetes mellitus and all five risk factors withing target range had an HR at 0.56 (95% CI 0.35–0.89).

Fig. 4 Panel E–H, shows the relative importance of each variable to the models associated with each of the outcomes in persons with type 2 diabetes mellitus. For atherosclerotic peripheral arterial disease, blood pressure, smoking and HbA1c showed the greatest relative importance, whereas blood pressure, smoking and HbA1c were the most important factors for aortic disease. Smoking, SBP and DBP explained roughly 30% each for all outcomes, whereas HbA1c explained between 10 and 20% of the models (Fig. 4 Panel E–H). Relative importance models for the entire cohort (i.e., all time periods) shows that SBP, smoking, HbA1c and duration of diabetes were the most important for peripheral atherosclerotic disease. Similar predictors were important for aortic disease, with the exception of aortic dissection, where smoking was not as important. For diabetic foot disease, the model was dominated by HbA1c and duration of diabetes (Supplementary Fig. S4). Competing risk regression was also performed to validate results. Increasing levels for SBP was also associated with a clear risk reduction, whereas an increase of DBP showed similar results as cause-specific Cox models (Supplementary Fig. S5).

### Diabetic foot disease

Fig. 5 Panel A–E, shows results for all principal analyses regarding diabetic foot disease in persons with type 2 diabetes mellitus and matched controls. Fig. 5 Panel A shows that incidence rates for diabetic foot disease have decreased over two decades from 309.8 to 226.8 events per 100,000 person-years in persons with type 2 diabetes mellitus. Lower extremity artery disease and diabetic foot disease are the most common peripheral arterial complications in persons with type 2 diabetes mellitus, and diabetic foot disease has become the most prevalent of all peripheral arterial complications. Results from Kaplan–Meier survival curves show that after incident diagnosis of diabetic foot disease, less than 25% of all study participants had survived after 10-years of follow-up (Fig. 5 Panel B). Lower HbA1c, SBP and TG levels were associated with reduced risk for diabetic foot disease, and increasing levels of HbA1c and SBP showed a marked risk increase (Fig. 5 Panel C). HbA1c and duration of diabetes together explained 60% of the Cox model for diabetic foot disease (Fig. 5 Panel D). Individuals with type 2 diabetes mellitus that had one risk factor out of target range displayed no excess risk of diabetic foot disease (HR 1.10, 95% CI 0.97–1.24) and those patients that had all risk factors at baseline had 3 times greater risk (HR 3.01, 95% CI 2.58–3.50), compared with individuals with type 2 diabetes mellitus and no risk factors at baseline (Fig. 5 Panel E).

### Thoracic- and abdominal aortic aneurysm

Supplementary Fig. S5 illustrates that thoracic aortic aneurysm is increasing, particularly among patients with type 2 diabetes mellitus, where the incidence has increased from 1.3 to 24.8 per 100,000 person-years, between the initial and last time period (Supplementary Fig. S5 Panel A). Abdominal aortic aneurysm remains relatively unchanged in type 2 diabetes mellitus, while it is decreasing steadily in matched controls. Optimal risk factor levels and relative variable importance shows fairly similar patterns for both thoracic- and abdominal aortic aneurysm.

## Discussion

Our results shows that patients with type 2 diabetes mellitus and matched controls from the general population experienced a significant reduction in incidence of atherosclerotic non-coronary peripheral arterial disease, as well as, diabetic foot disease for patients with type 2 diabetes mellitus. The incidence of thoracic aortic aneurysm has risen in both patients with type 2 diabetes mellitus and matched controls. In people with diabetes, abdominal aortic aneurysm increased until 2012, followed by a gradual decline, while controls experienced a continuous decrease in abdominal aortic aneurysm throughout the follow-up period. The incidence of aortic dissection remained relatively stable across both groups, during the entire study period. The results also suggest that patients with type 2 diabetes mellitus have a substantial risk reduction of aortic disease, even in patients with poor risk factor control.^19^ Incidence rates of diabetic foot disease has decreased significantly among patients with type 2 diabetes mellitus, but this condition has also emerged as the most common peripheral arterial complication in persons with type 2 diabetes mellitus. Overall survival probability after onset of extracranial larger artery disease and aortic diseases was significantly higher in patients with type 2 diabetes mellitus, compared to controls, which could indicate that patients with diabetes are identified early in the disease process, and that onset of extracranial large artery disease or aortic disease in matched controls is more sudden and associated with increased risk of all-cause mortality. More than half to three-quarters of all study participants who developed a peripheral arterial complication succumbed within ten years of follow-up. Comparing the present findings with previous publications on traditional cardiovascular disease in patients with type 2 diabetes mellitus from the same cohort, reveals a slower rate reduction over time for lower extremity artery disease and diabetic foot disease, compared with cardiovascular disease.^20^ Results from the present analyses shows relatively similar rate reduction, patterns for risk factor associations and relative risk factor contributions among people with type 2 diabetes mellitus with extracranial large artery disease and lower extremity artery disease.

Results from the relative importance analyses highlight the relative contribution between cardiometabolic risk factors and development of peripheral arterial complications. Dysglycemia, duration of diabetes (a proxy of dysglycemia), smoking and hypertension displayed strong relative contribution to all peripheral arterial complications whereas lipids only showed a strong relative contribution in the final time-period for extracranial large artery disease. The Cox models shows significantly lower risk associated with each level of HbA1c below therapeutic target levels for extracranial large artery disease, lower extremity artery disease and diabetic foot disease. In addition, multifactorial risk factor control analyses demonstrate a relatively small incremental risk association for each risk factor not within target range and the subsequent risk of outcomes. Altogether, these findings underscore the unique relationship between dysglycemia and peripheral arterial complications, particularly regarding atherosclerotic non-coronary peripheral arterial complications and diabetic foot disease.^4^^,^^21^, ^22^, ^23^ Comparing the present results to previous findings associating type 2 diabetes mellitus and with cardiovascular disease risk, the present results reveal that the relative associations between specific risk factors and sub-types of peripheral arterial complications vary. Increasing levels of SBP were linked to a lower risk of aortic aneurysm. On the other hand, increasing levels of DBP, LDL-C, TG and higher HDL-C, were associated with an elevated risk for aortic disease.

Individuals with type 2 diabetes mellitus who maintain all modifiable risk factors within the desired target range do not have associated excess risk of atherothrombotic peripheral arterial disease.^24^, ^25^, ^26^ Risk and rates of aortic disease are considerably lower among individuals with type 2 diabetes mellitus, indicating a potential protective effect of type 2 diabetes mellitus against both dissection- and aneurysmal development. These associations are likely due to dysglycemia that most likely drives a fibrotic or calcific vascular remodeling, rather than sclerotic and lipid rich plaque process development. The present findings emphasize the importance of comprehensive risk factor management in patients with type 2 diabetes mellitus to mitigate the risk of peripheral arterial disease. The observed lower associated risk from increasing HbA1c levels for the development of aortic diseases in patients with type 2 diabetes mellitus should not be interpreted as a positive process but rather as an indirect confirmation of the dominant pathological vascular processes were fibrosis and calcification accelerate atherosclerotic development and impairing hemodynamic function, resulting in pathological vessel changes that are decreasing the risk of developing aortic diseases.

Previously published results suggest that individuals with type 2 diabetes mellitus and poor risk factor control had the highest risk for heart failure hospitalization, out of all cardiovascular diseases. Results from the present study reveal that the excess risk for lower extremity artery disease is the highest among all cardiovascular conditions, including heart failure.^5^ It is noteworthy, however, that the cohort did not exclude patients with any form of cardiovascular disease (i.e., acute myocardial infarction, coronary heart disease or stroke) at baseline.

### Limitations

Information on cardiometabolic data for the control participants was not available. The regression models including patients with diabetes, uses imputed baseline values of risk factors and this may be considered a limitation, particularly for variables such as blood pressure, but using imputed values is advantageous from a clinical perspective if data can be considered missing at random. Matched controls may have developed type 2 diabetes mellitus during follow-up; however, this is likely to have a minimal impact on the results. The current study design introduces an inherent selection bias due to the contribution of observation time by participants across multiple periods without events. This leads to a sequential accumulation of healthier individuals in each period, potentially creating a cohort that progressively consists of individuals at lower risk. The results of this study are model dependent and could vary with different approaches to statistical analyses, mainly due to heavy adjustment for covariates and regression modeling strategies. No distinction was made between persons with all or some risk factors within target ranges without any specific intervention and persons who were medically treated to attain target levels; therefore, this is not a study assessing control of risk factors but instead having risk factors within range. No adjustments were made for multiple testing. It is also acknowledged that residual confounding is impossible to fully overcome.

### Conclusions

This study contributes information on the changing epidemiology of non-coronary peripheral arterial complications in persons with type 2 diabetes mellitus. The incidence of extracranial large artery disease, aortic dissection, lower extremity artery disease and diabetic foot disease have declined over the past two decades in those with and without type 2 diabetes mellitus, whereas aortic aneurysm appears to be increasing in recent years for people with type 2 diabetes mellitus. The risk of all-cause death after onset of peripheral arterial complications is substantially increased. The incremental risk of type 2 diabetes mellitus for each outcome assessed is increased in a stepwise fashion by the increasing number of cardiovascular risk factors that are beyond target levels. Compared with those without diabetes, individuals with type 2 diabetes mellitus and all risk factor within target levels, displayed virtually no excess risk for peripheral arterial complications, whereas type 2 diabetes mellitus was associated with reduced risk of aortic disease, regardless of risk factor control at baseline. Cardiometabolic risk factor pattern between peripheral arterial complications differs from cardiovascular disease, in persons with type 2 diabetes mellitus. Smoking, blood pressure and glycated hemoglobin displayed a much greater importance for disease development in the peripheral arterial tree, whereas lipids had a small relative contribution.

## Contributors

The study was conceptualized by the last author (Ai.R). The first two authors (Ar.R, B.E), as well as the last author (Ai.R) had full access to the data. The methodology, formal analysis, as well as, preparation of figures and tables were performed by the last authors (Ai.R). Ai.R. is the guarantor of this work and, as such, had all access to all the data in the study and takes responsibility for the integrity of the data and the accuracy of the data analysis. The first draft of the manuscript was written by the first author (Ar.R).

All of the authors participated in data analysis and interpretation. All authors vouch for the accuracy and completeness of the data and analyses, and made the decision to submit the manuscript for publication. All named authors meet the International Committee of Medical Journal Editors criteria for authorship for this article, take responsibility for the integrity of the work as a whole, and have given their approval for this version to be published.

## Data sharing statement

Because of the sensitive nature of the data collected for this study, access to the datasets is available from the sources stated in the paper on request to the data providers, fulfilling the legal and regulatory requirements, and with approval from the Swedish Ethical Review Authority.

## Declaration of interests

Dr. Bhatt discloses the following relationships - Advisory Board: Angiowave, Bayer, Boehringer Ingelheim, CellProthera, Cereno Scientific, Elsevier Practice Update Cardiology, High Enroll, Janssen, Level Ex, McKinsey, Medscape Cardiology, Merck, MyoKardia, NirvaMed, Novo Nordisk, PhaseBio, PLx Pharma, Stasys; Board of Directors: American Heart Association New York City, Angiowave (stock options), Bristol Myers Squibb (stock), DRS.LINQ (stock options), High Enroll (stock); Consultant: Broadview Ventures, GlaxoSmithKline, Hims, SFJ, Youngene; Data Monitoring Committees: Acesion Pharma, Assistance Publique-Hôpitaux de Paris, Baim Institute for Clinical Research (formerly Harvard Clinical Research Institute, for the PORTICO trial, funded by St. Jude Medical, now Abbott), Boston Scientific (Chair, PEITHO trial), Cleveland Clinic, Contego Medical (Chair, PERFORMANCE 2), Duke Clinical Research Institute, Mayo Clinic, Mount Sinai School of Medicine (for the ENVISAGE trial, funded by Daiichi Sankyo; for the ABILITY-DM trial, funded by Concept Medical; for ALLAY-HF, funded by Alleviant Medical), Novartis, Population Health Research Institute; Rutgers University (for the NIH-funded MINT Trial); Honoraria: American College of Cardiology (Senior Associate Editor, Clinical Trials and News, ACC.org” title = “http://ACC.org”>ACC.org; Chair, ACC Accreditation Oversight Committee), Arnold and Porter law firm (work related to Sanofi/Bristol-Myers Squibb clopidogrel litigation), Baim Institute for Clinical Research (formerly Harvard Clinical Research Institute; RE-DUAL PCI clinical trial steering committee funded by Boehringer Ingelheim; AEGIS-II executive committee funded by CSL Behring), Belvoir Publications (Editor in Chief, Harvard Heart Letter), Canadian Medical and Surgical Knowledge Translation Research Group (clinical trial steering committees), CSL Behring (AHA lecture), Cowen and Company, Duke Clinical Research Institute (clinical trial steering committees, including for the PRONOUNCE trial, funded by Ferring Pharmaceuticals), HMP Global (Editor in Chief, Journal of Invasive Cardiology), Journal of the American College of Cardiology (Guest Editor; Associate Editor), K2P (Co-Chair, interdisciplinary curriculum), Level Ex, Medtelligence/ReachMD (CME steering committees), MJH Life Sciences, Oakstone CME (Course Director, Comprehensive Review of Interventional Cardiology), Piper Sandler, Population Health Research Institute (for the COMPASS operations committee, publications committee, steering committee, and USA national co-leader, funded by Bayer), WebMD (CME steering committees), Wiley (steering committee); Other: Clinical Cardiology (Deputy Editor); Patent: Sotagliflozin (named on a patent for sotagliflozin assigned to Brigham and Women’s Hospital who assigned to Lexicon; neither I nor Brigham and Women’s Hospital receive any income from this patent); Research Funding: Abbott, Acesion Pharma, Afimmune, Aker Biomarine, Alnylam, Amarin, Amgen, AstraZeneca, Bayer, Beren, Boehringer Ingelheim, Boston Scientific, Bristol-Myers Squibb, Cardax, CellProthera, Cereno Scientific, Chiesi, CinCor, Cleerly, CSL Behring, Eisai, Ethicon, Faraday Pharmaceuticals, Ferring Pharmaceuticals, Forest Laboratories, Fractyl, Garmin, HLS Therapeutics, Idorsia, Ironwood, Ischemix, Janssen, Javelin, Lexicon, Lilly, Medtronic, Merck, Moderna, MyoKardia, NirvaMed, Novartis, Novo Nordisk, Otsuka, Owkin, Pfizer, PhaseBio, PLx Pharma, Recardio, Regeneron, Reid Hoffman Foundation, Roche, Sanofi, Stasys, Synaptic, The Medicines Company, Youngene, 89Bio; Royalties: Elsevier (Editor, Braunwald’s Heart Disease); Site Co-Investigator: Abbott, Biotronik, Boston Scientific, CSI, Endotronix, St. Jude Medical (now Abbott), Philips, SpectraWAVE, Svelte, Vascular Solutions; Trustee: American College of Cardiology; Unfunded Research: FlowCo. JN reports no conflicts of interest. Dr. McGuire reports research support for Clinical Trials Leadership from Boehringer Ingelheim, Merck & Co, Pfizer, AstraZeneca, Novo Nordisk, Esperion, Lilly USA, Lexicon, CSL Behringm New Amsterdam; honoraria for consultancy from Lilly USA, Boehringer Ingelheim, Merck & Co, Novo Nordisk, Applied Therapeutics, Altimmune, CSL Behring, Bayer, GlaxoSmithKline, Intercept. Dr. Eliasson reports personal fees from Amgen, AstraZeneca, Boehringer Ingelheim, Eli Lilly, Merck Sharp and Dohme, Mundipharma, NovoNordisk and Sanofi, all outside the submitted work. Dr. Borén reports personal fees from Novartis, Pfizer, Novo Nordisk, Amgen and Akcea. Dr. Sattar reports personal fees from Amgen, AstraZeneca, Boehringer Ingelheim, Eli Lilly, Merck Sharp & Dohme, Novartis, Novo Nordisk, Pfizer, Sanofi, Roche Diagnostics, Abbot Laboratories, AbbVie, Hanmi Pharmaceuticals, Janssen, Menarini-Richerche. Dr. Gerstein reports personal fees and grants from Sanofi, Eli Lilly, Novo Nordisk, Merck, Abbott, Hanmi, Boehringer Ingelheim, Carbon Bran, Bioling, AstraZZeneca, Zuellig, DKSH, Jiangsu Hanson Pharma and Kowa Research Institute. Dr. Beckman has received grants from Bristol Myers Squibb and consulting fees from JanOne. The remaining authors have nothing to disclose.
