# Supplementary material for: Non-coronary peripheral arterial complications in people with type 2 diabetes: a Swedish retrospective cohort study
Source: Lancet Reg Health Eur. 2024 Mar 19;39:100888. doi: 10.1016/j.lanepe.2024.100888 (PMC11129337; doi:10.1016/j.lanepe.2024.100888)
Supplement: Supplementary Material [file mmc1.pdf]

SUPPLEMENTAL MATERIAL

SUPPLEMENTAL MATERIAL 1

Statistical methods 3

Incidence rates and change in risk over time 3

Optimal levels for risk factors 3

Multifactorial risk factor control 4

Relative variable importance 4

Table S1. ICD-10 Codes Used to Define Baseline Conditions and Outcomes 5

Supplemental tables 5

Table S2. Variables Used in the Imputation Algorithm. 6

Table S3. Baseline characteristics for individuals with type 2 diabetes according to time-period for inclusion in the registry. 7

Table S4. Crude- and standardized incidence rates for peripheral arterial complications among both patients with diabetes and matched controls 10

Table S5. Excess risk of outcomes with adjustment for age, sex and socioeconomic variables in patients with type 2 diabetes 14

Supplementary Figure S1: Flowchart of study cohort and analyses 15

Supplemental figures 15

Supplementary Figure S2: Kaplan-Meier curves for peripheral arterial complications 16

Supplementary Figure S3: Competing risk regression and aortic complications 17

Supplementary Figure S4: Relative importance for peripheral arterial complications in the entire cohort 18

Supplementary Figure S5: Analyses of incidence rates, optimal levels for HbA1c, SBP and LDL–C, as well as, variable importance for thoracic- and abdominal aortic aneurysms 19

Statistical methods

We calculated crude- and standardized rates for cardiovascular complications, among patients with type 2 diabetes and controls. Cox proportional hazards models were used to assess the relative risk associated with the number of risk factors at target for patients with diabetes, compared to matched controls, and to assess optimal levels for selected risk factors, and estimation of relative variable importance from the Cox model.

Incidence rates and change in risk over time

Age- and sex standardized incidence rates were applied together with Swedish population life tables, from the initial time period as the reference population for type 2 diabetes case control cohort. We constructed the following age-categories, **1. < 45, 2. 45–54, 3. 55–64, 4. 65–74 and 5. > 75** years of age, for the reference population and our study cohort. The reference population was also stratified on sex.

Cox regression was used to estimate change in risk over time for various time-periods. These Cox models were adjusted for age and sex, as well as, time-period (i.e., P1–P9), which was modeled as an ordinal factor variable with P1 as the reference value. A similar model was constructed for supplementary figure S3.

Optimal levels for risk factors

We used Cox regression with restricted cubic splines and defined guideline target levels as reference level for each selected risk factor. For glycated hemoglobin we used 52 mmol/mol (7.0%), for systolic blood pressure 130 mmHg or diastolic blood pressure 80 mmHg, and for low-density lipoprotein cholesterol 2.5 mmol/L, for body mass index 27.5 kg/m^2, for eGFR 90 ml/min/1.73 m^2, total-cholesterol 5 mmol/L, triglycerides 1.7 mmol/L, high-density lipoprotein cholesterol 1.5 mmol/L. For continuous variables, we used restricted cubic splines with three evenly spaced knots.

These models were also adjusted for comorbidities, socioeconomic variables and pharmacological medications. The following R code provides an example of the modeling approach for lower extremity arterial disease (LEAD). A similar Cox model was constructed for all outcomes.

Example of R code:

$$\begin{aligned}
COX_LEAD <- cph(&Surv(survtime, event) \sim rcs(age, 3) + rcs(hba1c, 3) \\
&+ rcs(BMI, 3) + rcs(SBP, 3) + rcs(DBP, 3) + rcs(eGFR, 3) \\
&+ rcs(HDL, 3) + rcs(LDL, 3) + rcs(durationofdiabetes, 3) \\
&+ rcs(Triglycerides, 3) + (Smoker) + Physicalactivity \\
&+ strat(Sex) + strat(baseline_stroke) \\
&+ strat(baseline_heartfailure) + strat(baseline_hypertension) \\
&+ strat(baseline_cad) + strat(baseline_copd) \\
&+ strat(baseline_dementia) + strat(baseline_esrd) \\
&+ strat(baseline_cancer) + Maritalstatus + Education \\
&+ Ethnicity + income_IQR + strat(bloodpressure_med) \\
&+ strat(statins_med) + strat(anticoagulation_med) \\
&+ strat(antithrombotic_med), data = data, ties = "efron", x = T)
\end{aligned}$$

Multifactorial risk factor control

Using Cox regression that includes a covariate with number of risk factors at target ranging from 0 to 5 (referred to as $\boldsymbol{\beta_1(category)}$), modeled as categorical variable. For this variable, we included traditional and modifiable cardiometabolic risk factors such as, glycated hemoglobin, systolic- and diastolic blood pressure, low-density lipoprotein cholesterol, smoking and renal function (albuminuria). Matched controls had a duration of diabetes set to zero years and were also considered the reference *group* for the $\boldsymbol{\beta_1(category)}$ variable, whereas patients with diabetes had their duration of diabetes subtracted with the grand mean of the type 2 diabetes cohort. We also stratified the models for sex.

We also stratified this model for several of the categorical variables there included in the model. All Cox models are adjusted for age, sex, socioeconomic variables, baseline comorbidities and treatment with either antihypertensives, statins, antithrombotic- or anticoagulant medication.

Example of R Code:
The variables termed "Category" denotes the number of risk factors within target range at baseline, ranging from 0-5, with 0 being the reference value (i.e., controls).

$$\begin{aligned} COX\_LEAD \; < - \; & coxph(Surv(survtime, event) \sim Category + age \\ & + durationofdiabetes + strata(Sex) + income\_IQR \\ & + factor(Education) + factor(Maritalstatus) + factor(Ethnicity) \\ & + factor(baseline\_heartfailure) + factor(baseline\_cad) \\ & + factor(baseline\_stroke) + factor(baseline\_copd) \\ & + factor(baseline\_dementia) + factor(baseline\_hypertension) \\ & + factor(baseline\_esrd) + factor(baseline\_cancer) \\ & + strat(bloodpressure\_med) + strat(statins\_med) \\ & + strat(anticoagulation\_med) + strat(antithrombotic\_med), \\ & \qquad data = data, ties = "efron") \end{aligned}$$

Relative variable importance

Using an extended application for the Cox regression model with restricted cubic splines, we assessed the relative variable importance for modifiable risk factors and cardiovascular complications by estimating the partial effect, i.e., effect of each risk factor in terms of predicting the outcome. Log likelihood is used for quantifying the predictive information contained in a predictor, compared with the information contained in the model with predictors. The regression model for these analyses were almost identical with the Cox model for optimal cardiometabolic risk factor levels, with one exception, age was used as the time-scale since it would otherwise dominate the importance plot.

The partial effect of each risk factor was quantified by computing the proportion of explainable log-likelihood explained by each risk factor, i.e., the Wald X2 statistics minus the degrees of freedom (Harrell F. Regression Modeling Strategies. Springer; 2015).

Supplemental tables

Table S1. ICD-10 Codes Used to Define Baseline Conditions and Outcomes		
**Outcomes**		**ICD-10**
Lower extremity arterial disease		I702, I702A, I702C, I702D, I702X, I702E, I739B,1700
Diabetic foot disease		E115, E115A, E115B, E115W, E115X, E1151, E116D
Extracranial large artery disease		I630, I631, I632, I635, I652, I650, I659, I653, I658
Aortic aneurysm		I714, I719, I713, I712, I711, I716, I715, I718
Aortic dissection		I710B, I710, I710X, I710W, I710A
Thoracic aortic aneurysm		I712, I711, I716
Abdominal aortic aneurysm		I713, I714
Hypertension		I109, I11, I120, I129, I132, I139, I130, I131, I159, I150, I152, I158
Dementia		F00, F01, F02, F039 N179, N178, N170, N172, N189, N181, N182, N183, N184, N185, N199, Z940, DR016, N083, E112C,
End-stage renal disease		E112C, E112W, E112X, E102, E102X, E132, E242, E122, E112A, E112B, I120, Z992, DR013, DR023, DR056, Z941, Z492
Cancer		C00–C97
COPD		J44
Atrial fibrillation		I489, I480, I481, I482, I483, I483
Heart failure		I50
Coronary artery disease		I20-I25
Acute myocardial infarction		I214, I219, I210, I211, I213, I212)
Cerebrovascular disease		I61-I64
* Includes ICD-codes as main diagnosis and up to 6 contributory causes for outcomes and comorbidities		

Table S2. Variables Used in the Imputation Algorithm.
Age, sex, age at onset of diabetes, clinicians diagnosis type, epidemiological definition of diabetes, treatment of diabetes, method of insulin treatment, systolic blood pressure, diastolic blood pressure, body weight, body length, glycated hemoglobin (HbA1c), total cholesterol, triglycerides, HDL-cholesterol, LDL-cholesterol, albuminuria, s-creatinine, retinopathy, smoking status, physical activity, county, body mass index, marital status, education, ethnicity, income, family income, eGFR, history of acute myocardial infarction, coronary heart disease, heart failure, hypertension, peripheral arterial disease, chronic obstructive pulmonary disease, dementia, alcoholism, end-stage renal disease, cancer, stroke, atrial fibrillation, treatment with anti-hypertensive medication, statins, antithrombotic and anti-coagulant medication.

Table S3. Baseline characteristics for individuals with type 2 diabetes according to time-period for inclusion in the registry.

Time-Period	2001-2002	2003-2004	2005-2006	2007-2008	2009-2010	2011-2012	2013-2014	2015-2016	2017-2019
n	37165	53221	69920	89024	97089	73283	63804	67898	103846
Sex = female %)	17039 (45.8)	24192 (45.5)	31316 (44.8)	39456 (44.3)	43019 (44.3)	31587 (43.1)	26858 (42.1)	28403 (41.8)	45027 (43.4)
Age (mean (SD))	66.12 (12.82)	65.85 (12.35)	65.55 (12.30)	65.17 (12.40)	64.66 (12.93)	63.83 (13.12)	63.21 (13.15)	62.97 (13.11)	62.46 (11.00)
Age-category (%)									
<45	2069 (5.6)	2697 (5.1)	3782 (5.4)	5162 (5.8)	6603 (6.8)	5726 (7.8)	5288 (8.3)	5873 (8.6)	6567 (6.3)
45-54	4506 (12.1)	6531 (12.3)	8623 (12.3)	11562 (13.0)	13528 (13.9)	10847 (14.8)	10191 (16.0)	11074 (16.3)	11622 (11.2)
55-64	9701 (26.1)	14706 (27.6)	19811 (28.3)	25254 (28.4)	26728 (27.5)	19807 (27.0)	17218 (27.0)	18400 (27.1)	51169 (49.3)
65-74	10137 (27.3)	15044 (28.3)	19734 (28.2)	25414 (28.5)	27619 (28.4)	21595 (29.5)	18721 (29.3)	19967 (29.4)	20847 (20.1)
>75	10752 (28.9)	14243 (26.8)	17970 (25.7)	21632 (24.3)	22611 (23.3)	15308 (20.9)	12386 (19.4)	12584 (18.5)	13641 (13.1)
Education n (%)									
Pre-secondary education ≤ 9 years	19733 (53.1)	26412 (49.6)	32065 (45.9)	36985 (41.5)	37503 (38.6)	26088 (35.6)	21461 (33.6)	21162 (31.2)	32742 (31.5)
Secondary education >9 to 12 years	13284 (35.7)	20223 (38.0)	28533 (40.8)	38048 (42.7)	42718 (44.0)	33087 (45.1)	29582 (46.4)	32181 (47.4)	48919 (47.1)
Post-secondary education ≥ 12 years	4148 (11.2)	6586 (12.4)	9322 (13.3)	13991 (15.7)	16868 (17.4)	14108 (19.3)	12761 (20.0)	14555 (21.4)	22185 (21.4)
Civil status: married n (%)	20203 (54.4)	28984 (54.5)	37267 (53.3)	46660 (52.4)	49542 (51.0)	37050 (50.6)	31720 (49.7)	33841 (49.8)	52362 (50.4)
Ethnicity = Scandinavian, n (%)	34471 (92.8)	48790 (91.7)	62595 (89.5)	76858 (86.3)	80991 (83.4)	61026 (83.3)	51571 (80.8)	53748 (79.2)	80216 (77.2)
Income family Quartiles n (%)									
Quartile 1	17785 (47.9)	21918 (41.2)	26464 (37.8)	29450 (33.1)	29807 (30.7)	20888 (28.5)	16613 (26.0)	16569 (24.4)	16595 (16.0)
Quartile 2	12032 (32.4)	18162 (34.1)	24394 (34.9)	28915 (32.5)	29476 (30.4)	21036 (28.7)	17069 (26.8)	17275 (25.4)	17074 (16.4)
Quartile 3	5417 (14.6)	9456 (17.8)	13119 (18.8)	18111 (20.3)	19813 (20.4)	15004 (20.5)	13891 (21.8)	14536 (21.4)	48052 (46.3)
Quartile 4	1931 (5.2)	3685 (6.9)	5943 (8.5)	12548 (14.1)	17993 (18.5)	16355 (22.3)	16231 (25.4)	19518 (28.7)	22125 (21.3)
Income Quartiles n (%)									
Quartile 1	17527 (47.2)	19960 (37.5)	21488 (30.7)	21218 (23.8)	19428 (20.0)	13370 (18.2)	9840 (15.4)	9739 (14.3)	17887 (17.2)
Quartile 2	9733 (26.2)	15702 (29.5)	22833 (32.7)	27655 (31.1)	28473 (29.3)	20117 (27.5)	15576 (24.4)	15380 (22.7)	23494 (22.6)
Quartile 3	7037 (18.9)	12107 (22.7)	16948 (24.2)	22559 (25.3)	24528 (25.3)	17563 (24.0)	16465 (25.8)	16996 (25.0)	25470 (24.5)
Quartile 4	2868 (7.7)	5452 (10.2)	8651 (12.4)	17592 (19.8)	24660 (25.4)	22233 (30.3)	21923 (34.4)	25783 (38.0)	36995 (35.6)
Heart failure n (%)	2288 (6.2)	2982 (5.6)	4465 (6.4)	5730 (6.4)	6429 (6.6)	4424 (6.0)	3767 (5.9)	3819 (5.6)	5655 (5.4)
Coronary heart disease n (%)	4740 (12.8)	7572 (14.2)	11299 (16.2)	14406 (16.2)	15528 (16.0)	10922 (14.9)	9147 (14.3)	9405 (13.9)	13824 (13.3)

**Hypertension n (%)**	4748 (12.8)	8414 (15.8)	15082 (21.6)	23590 (26.5)	29637 (30.5)	23479 (32.0)	21521 (33.7)	23113 (34.0)	35643 (34.3)
**Chronic obstructive pulmonary disease n (%)**	594 (1.6)	989 (1.9)	1592 (2.3)	2351 (2.6)	2864 (2.9)	2267 (3.1)	2122 (3.3)	2193 (3.2)	3370 (3.2)
**Dementia n (%)**	190 (0.5)	245 (0.5)	401 (0.6)	617 (0.7)	989 (1.0)	729 (1.0)	512 (0.8)	521 (0.8)	653 (0.6)
**End-stage kidney disease n (%)**	719 (1.9)	946 (1.8)	1441 (2.1)	1811 (2.0)	2211 (2.3)	1570 (2.1)	1471 (2.3)	1599 (2.4)	2602 (2.5)
**Cancer n (%)**	2260 (6.1)	4056 (7.6)	6260 (9.0)	8990 (10.1)	10766 (11.1)	8503 (11.6)	7735 (12.1)	8616 (12.7)	14136 (13.6)
**Antihypertensive medication n (%)**	13079 (35.2)	22777 (42.8)	34399 (49.2)	49576 (55.7)	57994 (59.7)	46874 (64.0)	42726 (67.0)	47124 (69.4)	72401 (69.7)
**Statins n (%)**	10924 (29.4)	19239 (36.1)	29058 (41.6)	41838 (47.0)	48593 (50.0)	39591 (54.0)	36364 (57.0)	40042 (59.0)	58655 (56.5)
**Antiocoagulant medication n (%)**	3142 (8.5)	5341 (10.0)	7848 (11.2)	10903 (12.2)	12173 (12.5)	9336 (12.7)	8172 (12.8)	8807 (13.0)	13352 (12.9)
**Antithrombotic medication n (%)**	6610 (17.8)	11276 (21.2)	16211 (23.2)	22185 (24.9)	23543 (24.2)	17115 (23.4)	14645 (23.0)	15188 (22.4)	21809 (21.0)
**Age at onset of diabetes (mean (SD))**	58.93 (13.14)	59.51 (12.64)	59.51 (12.69)	59.91 (12.64)	60.33 (13.10)	60.64 (13.18)	60.69 (13.27)	60.90 (13.20)	60.84 (13.37)
**Duration of diabetes (mean (SD))**	7.08 (7.69)	6.24 (6.93)	5.88 (6.78)	5.05 (6.39)	4.06 (5.91)	2.89 (5.12)	2.17 (4.48)	1.70 (3.96)	1.52 (3.71)
**Glycated hemoglobin levels (mean (SD))**	55.24 (14.39)	54.77 (13.92)	53.62 (13.81)	53.25 (14.37)	54.35 (15.48)	55.55 (17.56)	56.40 (19.12)	56.50 (19.08)	56.13 (19.38)
**Current smoking n (%)**	5087 (13.7)	7980 (15.0)	10540 (15.1)	13748 (15.4)	16278 (16.8)	12089 (16.5)	10534 (16.5)	11218 (16.5)	16414 (15.8)
**Albuminuria n (%)**									
**No albuminuria**	28117 (75.7)	41436 (77.9)	54073 (77.3)	69418 (78.0)	77125 (79.4)	59650 (81.4)	52559 (82.4)	56223 (82.8)	87237 (84.0)
**Normal albuminuria**	97 (0.3)	58 (0.1)	121 (0.2)	539 (0.6)	567 (0.6)	428 (0.6)	247 (0.4)	315 (0.5)	432 (0.4)
**Microalbuminuria**	5287 (14.2)	8126 (15.3)	10321 (14.8)	13042 (14.6)	13148 (13.5)	9410 (12.8)	8004 (12.5)	8528 (12.6)	12453 (12.0)
**Macroalbuminuria**	3664 (9.9)	3601 (6.8)	5405 (7.7)	6025 (6.8)	6249 (6.4)	3795 (5.2)	2994 (4.7)	2832 (4.2)	3724 (3.6)
**eGFR (mean (SD))**	76.71 (25.43)	75.79 (23.51)	80.46 (24.90)	83.01 (25.48)	84.15 (26.50)	86.48 (27.73)	87.85 (29.99)	88.22 (28.63)	88.98 (30.71)
**Retinopathy n (%)**	8598 (23.1)	11267 (21.2)	13729 (19.6)	16577 (18.6)	16776 (17.3)	11180 (15.3)	9161 (14.4)	9474 (14.0)	13371 (12.9)
**Systolic blood pressure (mean (SD))**	144.32 (19.25)	142.26 (18.27)	139.69 (17.63)	138.18 (17.12)	137.21 (17.01)	136.81 (16.87)	136.32 (16.67)	136.19 (16.57)	135.60 (16.34)
**Diastolic blood pressure (mean (SD))**	79.31 (9.72)	78.63 (9.55)	78.06 (9.71)	78.50 (9.73)	78.83 (9.79)	79.45 (9.98)	79.94 (10.08)	80.36 (10.17)	80.49 (10.08)
**Total cholesterol (mean (SD))**	198.16 (43.55)	198.07 (41.71)	193.88 (41.46)	193.28 (42.47)	196.22 (44.52)	198.91 (45.39)	198.30 (46.40)	197.81 (46.28)	196.09 (46.26)
**High-density lipoprotein cholesterol (mean (SD))**	51.98 (17.74)	51.44 (17.14)	51.63 (16.91)	49.93 (16.72)	48.90 (16.64)	48.72 (16.42)	47.82 (15.83)	47.54 (15.62)	47.14 (15.05)
**Triglycerides (mean (SD))**	178.39 (125.85)	177.45 (123.42)	172.92 (117.92)	173.63 (117.76)	179.14 (128.47)	183.65 (136.10)	187.45 (141.69)	186.58 (145.09)	186.85 (148.71)
**Low-density lipoprotein cholesterol (mean (SD))**	113.07 (37.31)	113.35 (36.27)	109.78 (35.62)	110.89 (36.43)	113.92 (37.73)	116.13 (38.42)	115.96 (38.67)	116.22 (38.87)	115.16 (39.06)
**S-creatinine (mean (SD))**	83.69 (30.91)	84.32 (28.09)	80.66 (28.57)	78.63 (27.54)	77.88 (27.59)	76.39 (26.87)	75.92 (26.79)	75.50 (25.85)	74.89 (23.59)
**Body mass index (mean (SD))**	29.40 (5.15)	29.54 (5.21)	29.74 (5.26)	29.95 (5.38)	30.26 (5.58)	30.47 (5.67)	30.68 (5.69)	30.85 (5.85)	30.96 (5.86)
**Physical activity n (%)**									
1=Never	6121 (16.5)	8845 (16.6)	12667 (18.1)	15042 (16.9)	17235 (17.8)	12934 (17.6)	11558 (18.1)	12937 (19.1)	18691 (18.0)

2=<1 time/week	4992 (13.4)	7177 (13.5)	8610 (12.3)	10769 (12.1)	13048 (13.4)	10000 (13.6)	8786 (13.8)	9861 (14.5)	14075 (13.6)
3= 1-2 times/week	7376 (19.8)	10539 (19.8)	15123 (21.6)	17624 (19.8)	19041 (19.6)	14443 (19.7)	12335 (19.3)	12710 (18.7)	19135 (18.4)
4= 3-5 times/week	8029 (21.6)	11348 (21.3)	14248 (20.4)	19472 (21.9)	20345 (21.0)	15931 (21.7)	13340 (20.9)	13764 (20.3)	21484 (20.7)
Daily	10647 (28.6)	15312 (28.8)	19272 (27.6)	26117 (29.3)	27420 (28.2)	19975 (27.3)	17785 (27.9)	18626 (27.4)	30461 (29.3)
**Insulin use = Yes (%)**	203 (0.6)	267 (0.5)	322 (0.5)	351 (0.4)	373 (0.4)	312 (0.4)	254 (0.4)	263 (0.4)	373 (0.4)

**Table S4. Crude- and standardized incidence rates for peripheral arterial complications among both patients with diabetes and matched controls**							
**Event / Period**	**Category**	**Events**	**Person-years**	**Crude rate**	**Adj rate**	**Adj - LCI**	**Adj - UCI**
ELAD / [2001,2002]	Diabetes	80	31862.8	251.1	170	112.7	283.4
ELAD / [2003,2004]	Diabetes	274	116413.7	235.4	134.3	108.3	175.7
ELAD / [2005,2006]	Diabetes	668	229475.4	291.1	157.5	139.6	183
ELAD / [2007,2008]	Diabetes	955	369641.9	258.4	128.1	116.4	144.6
ELAD / [2009,2010]	Diabetes	1238	529505.5	233.8	119.6	109.5	133.1
ELAD / [2011,2012]	Diabetes	1481	662221.6	223.6	115	105.6	126.9
ELAD / [2013,2014]	Diabetes	1532	741716.1	206.5	100.8	93.6	110.4
ELAD / [2015,2016]	Diabetes	1473	814528.3	180.8	85.1	78.8	93.5
ELAD / [2017,2019]	Diabetes	2435	1365401.7	178.3	84.9	79.5	91.5
ELAD / [2001,2002]	Controls	166	105241.9	157.7	79.9	67.8	102
ELAD / [2003,2004]	Controls	587	374560.3	156.7	84.3	76.3	95.1
ELAD / [2005,2006]	Controls	1188	734803.9	161.7	84	78.9	90.5
ELAD / [2007,2008]	Controls	1510	1197377.5	126.1	66.4	62.4	71.1
ELAD / [2009,2010]	Controls	2120	1766269.1	120	63.4	60.4	66.9
ELAD / [2011,2012]	Controls	2494	2299176.9	108.5	56.9	54.3	59.8
ELAD / [2013,2014]	Controls	2768	2688037.3	103	52.7	50.5	55.4
ELAD / [2015,2016]	Controls	2974	3092416.9	96.2	48.2	46.2	50.5
ELAD / [2017,2019]	Controls	5088	5525759.1	92.1	44.1	42.7	45.8
AA / [2001,2002]	Diabetes	32	31895.7	100.3	40.6	27.7	126.4
AA / [2003,2004]	Diabetes	101	116660.6	86.6	39.1	31.6	66.4
AA / [2005,2006]	Diabetes	243	230226.8	105.5	47.6	41.2	63.5

AA / [2007,2008]	Diabetes	489	371177.5	131.7	61.8	52.8	76.1
AA / [2009,2010]	Diabetes	881	531688.3	165.7	68.8	64.1	77.3
AA / [2011,2012]	Diabetes	1127	664850.1	169.5	71.2	66.8	78.5
AA / [2013,2014]	Diabetes	1158	744534.5	155.5	64.6	59.9	71.7
AA / [2015,2016]	Diabetes	1199	817452.4	146.7	61.3	56.4	68.4
AA / [2017,2019]	Diabetes	2096	1369390.9	153.1	69.2	64.4	75.2
AA / [2001,2002]	Controls	196	105238	186.2	96.3	82.9	119.5
AA / [2003,2004]	Controls	511	374719.1	136.4	69.2	63.1	78
AA / [2005,2006]	Controls	1009	735474	137.2	72.1	67	78.6
AA / [2007,2008]	Controls	1597	1198395.9	133.3	70	66.2	74.8
AA / [2009,2010]	Controls	2754	1767152.6	155.8	81.7	78.4	85.6
AA / [2011,2012]	Controls	3223	2299232.7	140.2	73.4	70.4	76.8
AA / [2013,2014]	Controls	3722	2687033	138.5	70.3	67.6	73.4
AA / [2015,2016]	Controls	4080	3090186.2	132	66	63.5	68.9
AA / [2017,2019]	Controls	7638	5518421.8	138.4	66.4	64.6	68.3
AD / [2001,2002]	Diabetes	5	31908.7	15.7	9.3	2.3	100.1
AD / [2003,2004]	Diabetes	8	116758.3	6.9	3.9	1.4	32.6
AD / [2005,2006]	Diabetes	20	230547.3	8.7	5.1	2.8	20.4
AD / [2007,2008]	Diabetes	24	371999.4	6.5	3.8	2.2	14
AD / [2009,2010]	Diabetes	47	533421.6	8.8	4.4	3.1	11.4
AD / [2011,2012]	Diabetes	66	667969.2	9.9	6.1	3.4	12.6
AD / [2013,2014]	Diabetes	69	748985.6	9.2	4.7	3.4	9.8
AD / [2015,2016]	Diabetes	77	823142.4	9.4	5.7	3.4	11.2

AD / [2017,2019]	Diabetes	158	1380088.7	11.4	5.6	4.6	8.5
AD / [2001,2002]	Controls	18	105312.5	17.1	9.7	5.7	28
AD / [2003,2004]	Controls	68	375152.1	18.1	9.8	7.5	15.8
AD / [2005,2006]	Controls	138	736611.5	18.7	10.6	8.4	14.5
AD / [2007,2008]	Controls	209	1200797	17.4	10.1	8.5	12.9
AD / [2009,2010]	Controls	320	1771756.8	18.1	10.3	8.9	12.4
AD / [2011,2012]	Controls	336	2307345.4	14.6	8.2	7.2	9.8
AD / [2013,2014]	Controls	432	2698850.6	16	8.7	7.7	10.1
AD / [2015,2016]	Controls	484	3105993.3	15.6	8.7	7.6	10.2
AD / [2017,2019]	Controls	931	5551145.4	16.8	8.8	8.1	9.8
LEAD / [2001,2002]	Diabetes	206	31785.1	648.1	338.8	278.1	448.9
LEAD / [2003,2004]	Diabetes	625	115999.6	538.8	267.3	244.1	304.5
LEAD / [2005,2006]	Diabetes	1154	228485	505.1	245.7	227	271.4
LEAD / [2007,2008]	Diabetes	1898	367928.6	515.9	247.6	233.5	266.3
LEAD / [2009,2010]	Diabetes	2817	526425.2	535.1	251.1	239.3	265.9
LEAD / [2011,2012]	Diabetes	3291	657614.3	500.4	235	224.2	248.1
LEAD / [2013,2014]	Diabetes	3419	735765.4	464.7	219.4	209.1	231.8
LEAD / [2015,2016]	Diabetes	3647	807368.7	451.7	215.7	204.5	228.9
LEAD / [2017,2019]	Diabetes	5685	1352539.4	420.3	190.8	184	198.7
LEAD / [2001,2002]	Controls	307	105154.5	292	155	132.5	186.8
LEAD / [2003,2004]	Controls	782	374290	208.9	104	96.6	114.1
LEAD / [2005,2006]	Controls	1508	734493.9	205.3	101.2	96	107.7
LEAD / [2007,2008]	Controls	2412	1196663.1	201.6	102.9	98.3	108.4

LEAD / [2009,2010]	Controls	3189	1764871.5	180.7	92.3	88.8	96.3
LEAD / [2011,2012]	Controls	3982	2297050.8	173.4	88.9	85.8	92.4
LEAD / [2013,2014]	Controls	4003	2685034.3	149.1	74.3	71.8	77.2
LEAD / [2015,2016]	Controls	4349	3089205.3	140.8	68.8	66.5	71.4
LEAD / [2017,2019]	Controls	7216	5519612.5	130.7	60.9	59.3	62.7
DFD / [2001,2002]	Diabetes	164	31809	515.6	309.8	246.9	422.9
DFD / [2003,2004]	Diabetes	510	116123.4	439.2	268.6	227.8	323.7
DFD / [2005,2006]	Diabetes	991	228795	433.1	269.7	238.8	308.2
DFD / [2007,2008]	Diabetes	1742	368412.2	472.8	296.7	269.6	328.7
DFD / [2009,2010]	Diabetes	2352	527202.5	446.1	280	258	305.3
DFD / [2011,2012]	Diabetes	2736	659100.6	415.1	250.2	232.1	270.8
DFD / [2013,2014]	Diabetes	3043	737597.2	412.6	249.6	232.2	269.4
DFD / [2015,2016]	Diabetes	3555	808951.6	439.5	260.1	244.3	278.1
DFD / [2017,2019]	Diabetes	5363	1354575.4	395.9	226.8	215.2	239.6

Table S5. Excess risk of outcomes with adjustment for age, sex and socioeconomic variables in patients with type 2 diabetes

Variables	ELAD			AA			AA			LEAD			DFD		
HR[1]	HR[1]	95% CI[1]	p-value	HR[1]	95% CI[1]	p-value	HR[1]	95% CI[1]	p-value	HR[1]	95% CI[1]	p-value	HR[1]	95% CI[1]	p-value
**Category**															
Controls	—	—		—	—		—	—		—	—				
Type 2 diabetes	1.65	1.62, 1.70	<0.001	0.89	0.86, 0.91	<0.001	0.52	0.47, 0.57	<0.001	2.51	2.46, 2.55	<0.001	N/A		
Age – years	1.06	1.06, 1.06	<0.001	1.06	1.06, 1.06	<0.001	1.05	1.04, 1.05	<0.001	1.07	1.07, 1.07	<0.001	1.04	1.04, 1.04	<0.001
Sex = Women %	0.58	0.57, 0.59	<0.001	0.30	0.29, 0.30	<0.001	0.54	0.51, 0.59	<0.001	0.68	0.67, 0.69	<0.001	0.50	0.49, 0.52	<0.001
**Ethnicity**															
All other countries	—	—		—	—		—	—		—	—		—	—	
Scandinavia	1.23	1.18, 1.29	<0.001	1.43	1.36, 1.49	<0.001	1.53	1.32, 1.76	<0.001	1.23	1.19, 1.27	<0.001	1.28	1.22, 1.34	<0.001
**Civil**															
All other marital statuses	—	—		—	—		—	—		—	—		—	—	
Married	0.99	0.96, 1.01	0.3	1.02	1.00, 1.04	0.064	1.04	0.97, 1.11	0.3	0.80	0.79, 0.82	<0.001	0.73	0.71, 0.75	<0.001
**Education**															
Post-secondary education ≥ 12 years	—	—		—	—		—	—		—	—		—	—	
Pre-secondary education ≤ 9 years	1.17	1.13, 1.21	<0.001	1.29	1.24, 1.33	<0.001	0.91	0.82, 1.00	0.053	1.42	1.38, 1.47	<0.001	1.15	1.10, 1.20	<0.001
Secondary education >9 to 12 years	1.14	1.10, 1.18	<0.001	1.25	1.21, 1.29	<0.001	1.00	0.92, 1.10	>0.9	1.38	1.34, 1.42	<0.001	1.11	1.06, 1.16	<0.001
**Income IQR**															
IQR 1	—	—		—	—		—	—		—	—		—	—	
IQR 2	1.00	0.98, 1.03	0.8	1.12	1.08, 1.15	<0.001	1.15	1.05, 1.26	0.002	0.97	0.95, 1.0	0.015	0.85	0.83, 0.88	<0.001
IQR 3	0.81	0.78, 0.84	<0.001	0.98	0.94, 1.01	0.2	0.95	0.85, 1.05	0.3	0.77	0.75, 0.80	<0.001	0.70	0.67, 0.73	<0.001
IQR 4	0.71	0.68, 0.74	<0.001	0.91	0.87, 0.94	<0.001	0.89	0.79, 1.00	0.048	0.65	0.63, 0.67	<0.001	0.63	0.60, 0.66	<0.001

[1] HR = Hazard Ratio, CI = Confidence Interval

Supplementary Figure S1: Flowchart of study cohort and analyses

Supplementary Figure S1: Flowchart

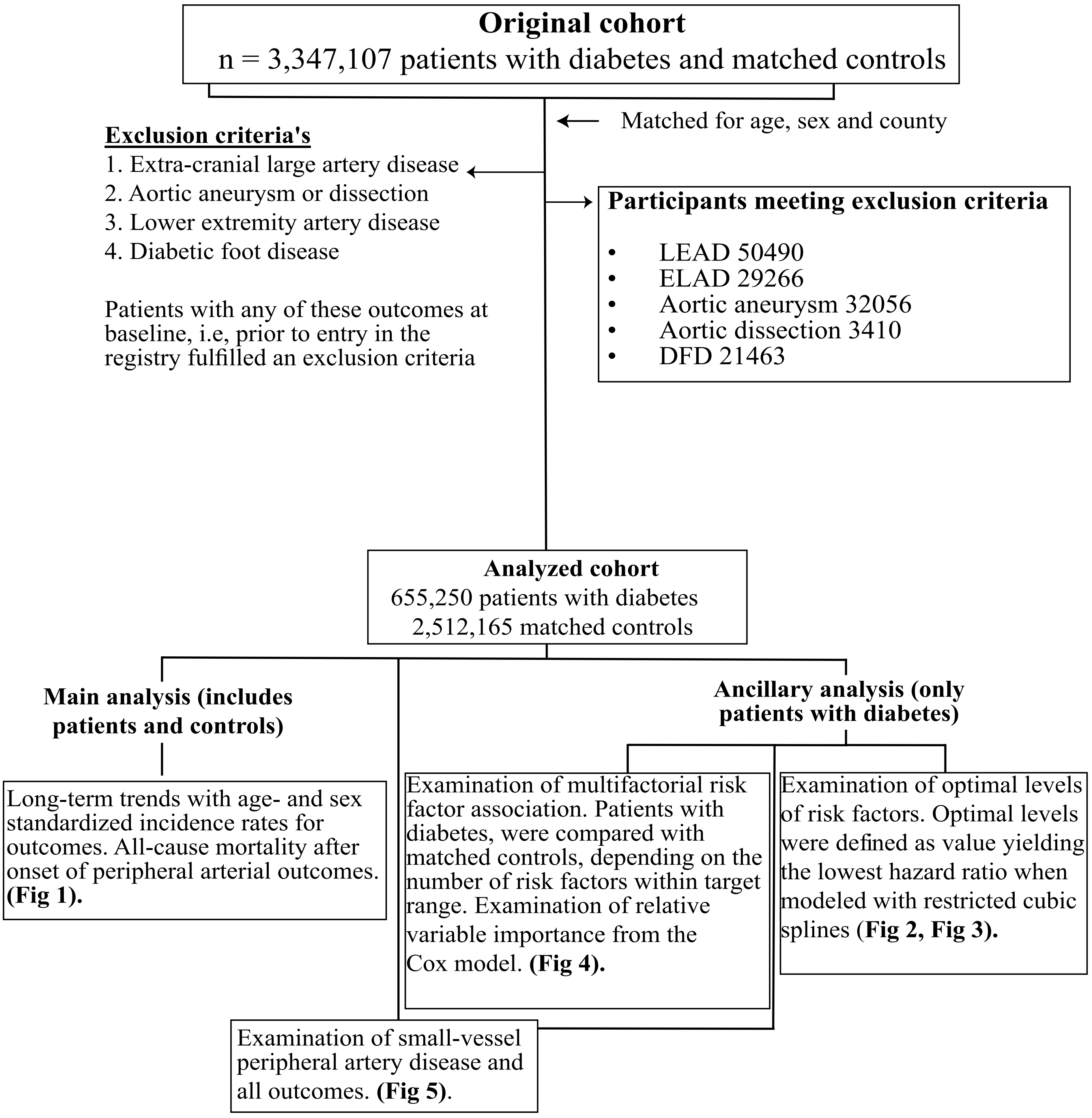

Legend: Participants may fulfill more than one exclusion criteria

Supplementary Figure S2: Kaplan-Meier curves for peripheral arterial complications

Supplemental Figure S2. Kaplan-Meier survival curves for all-cause mortality post onset of peripheral arterial complications

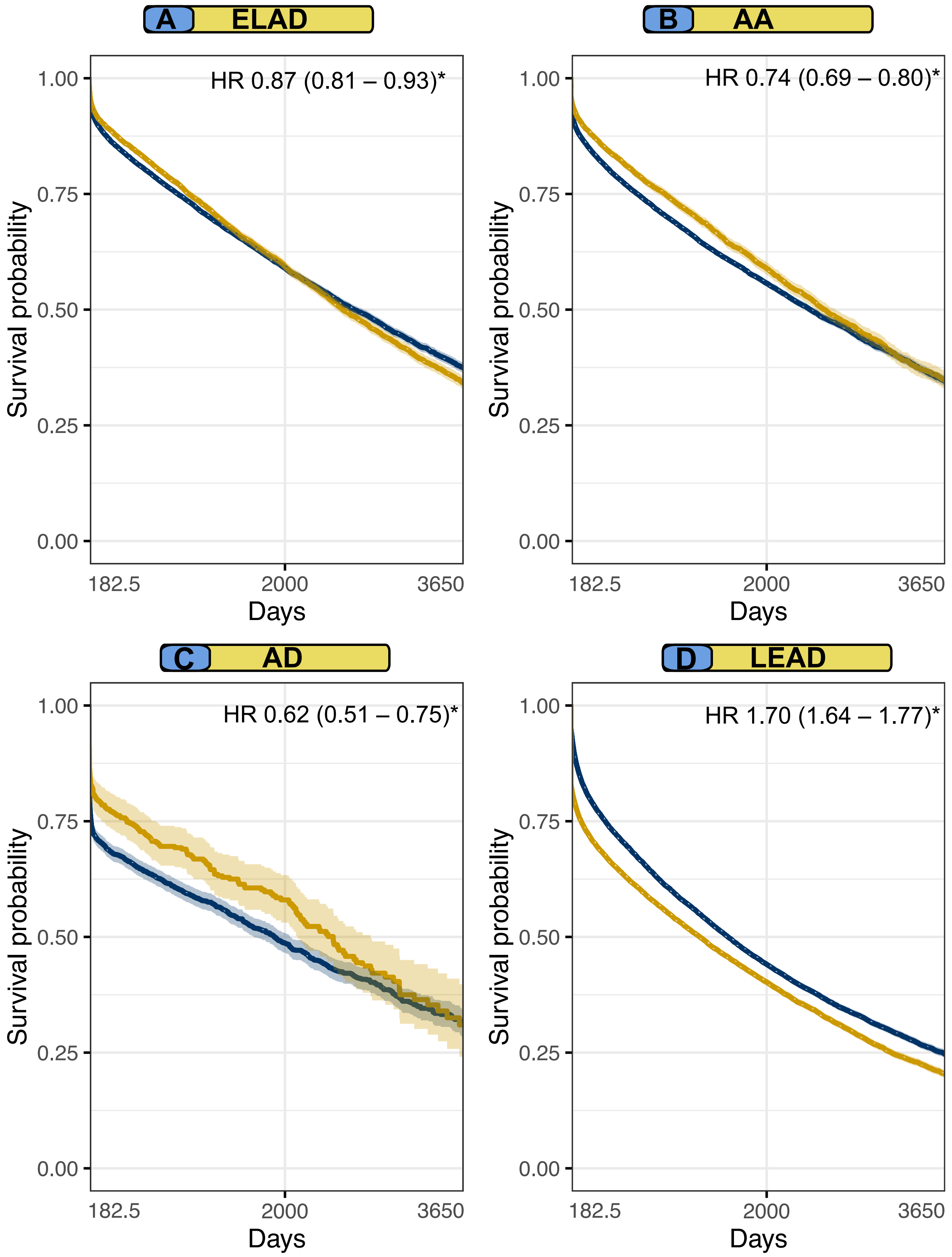

Figure legend: Kaplan-Meier curves between 6 months and 10 years of follow-up. The hazard ratios shows the risk for individuals with diabetes according to Cox regression, adjusted for age and sex.

Supplementary Figure S3: Competing risk regression and aortic complications

Supplemental Figure S3. Competing risk regression and hazard functions for risk factors for aortic aneurysm and dissection.

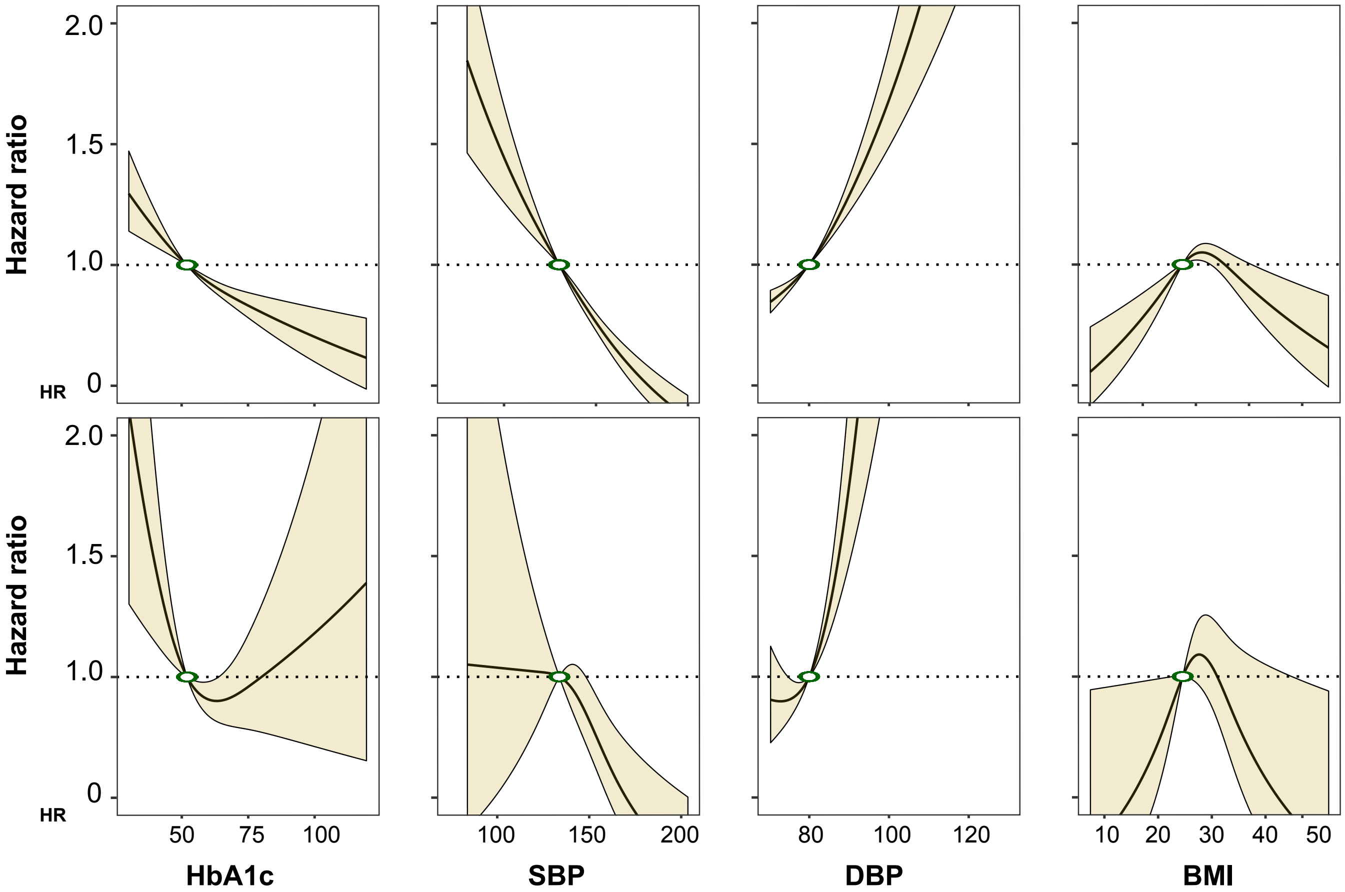

Figure legend: Cox models for glycated hemoglobin and blood pressure variables for aortic complications, using competing risk regression with all-cause mortality. Model construction is similar to Cox models in Figure 2 and Figure 3.

Excess risk for aortic outcomes in patients with T2D

HR and 95% CI	**AA**	**AD**
**Type 2 diabetes**	0.48 (0.47 - 0.50)	0.22 (0.19 - 0.25)
**Age**	0.972 (0.971 - 0.974)	0.932 (0.92 - 0.936)
**Sex**	0.61 (0.58 - 0.63)	0.83 (0.74 - 0.92)

These Cox models are adjusted for age, sex, pharmacological treatment and comorbidities

Supplementary Figure S4: Relative importance for peripheral arterial complications in the entire cohort

Supplemental Figure S4. Relative variable importance for peripheral arterial complications among patients with type 2 diabetes

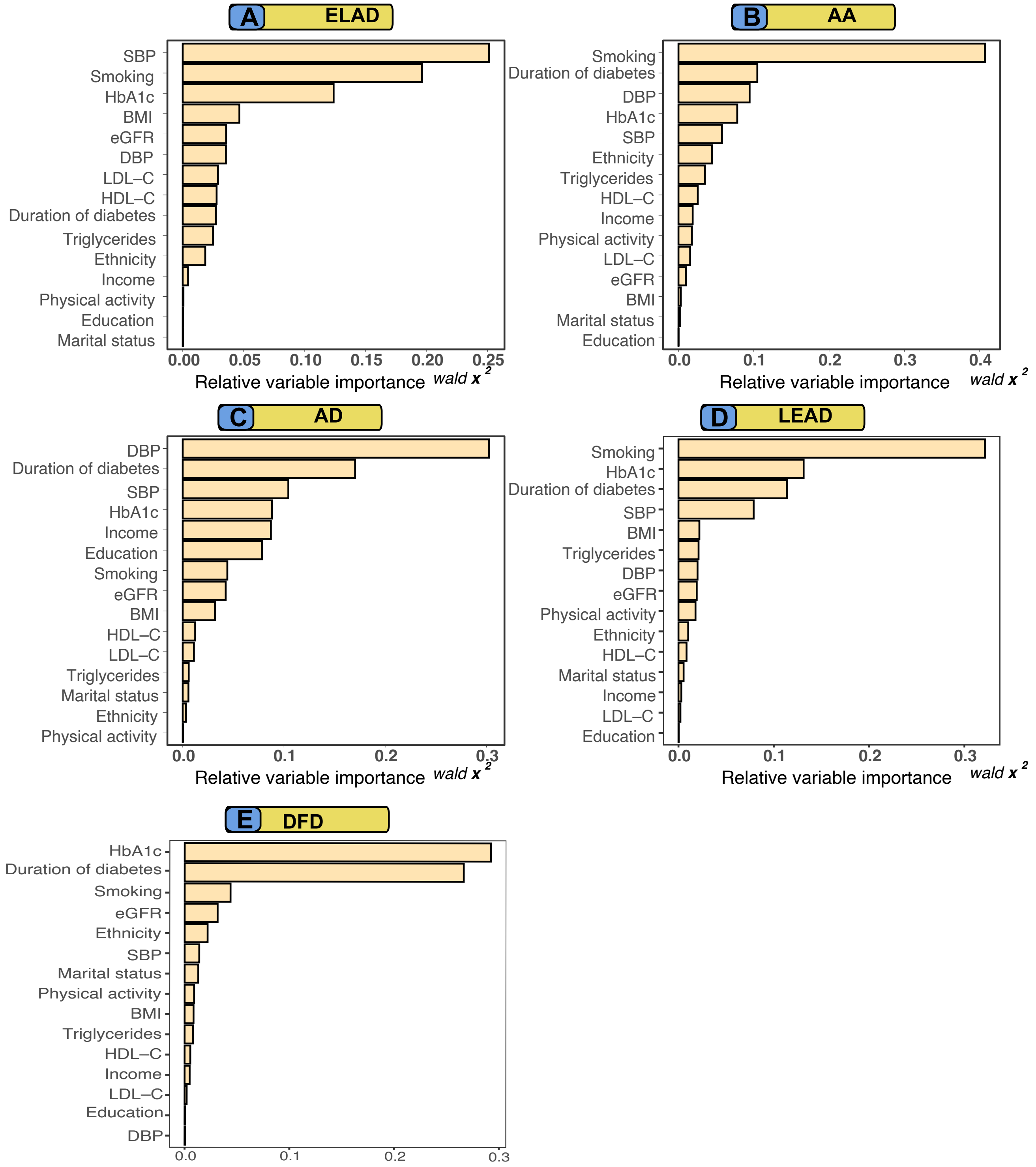

Figure legend: Variables with higher importance measures demonstrated a high predictive performance and are deemed important for modeling the outcome.

Supplementary Figure S5: Analyses of incidence rates, optimal levels for HbA1c, SBP and LDL–C, as well as, variable importance for thoracic- and abdominal aortic aneurysms

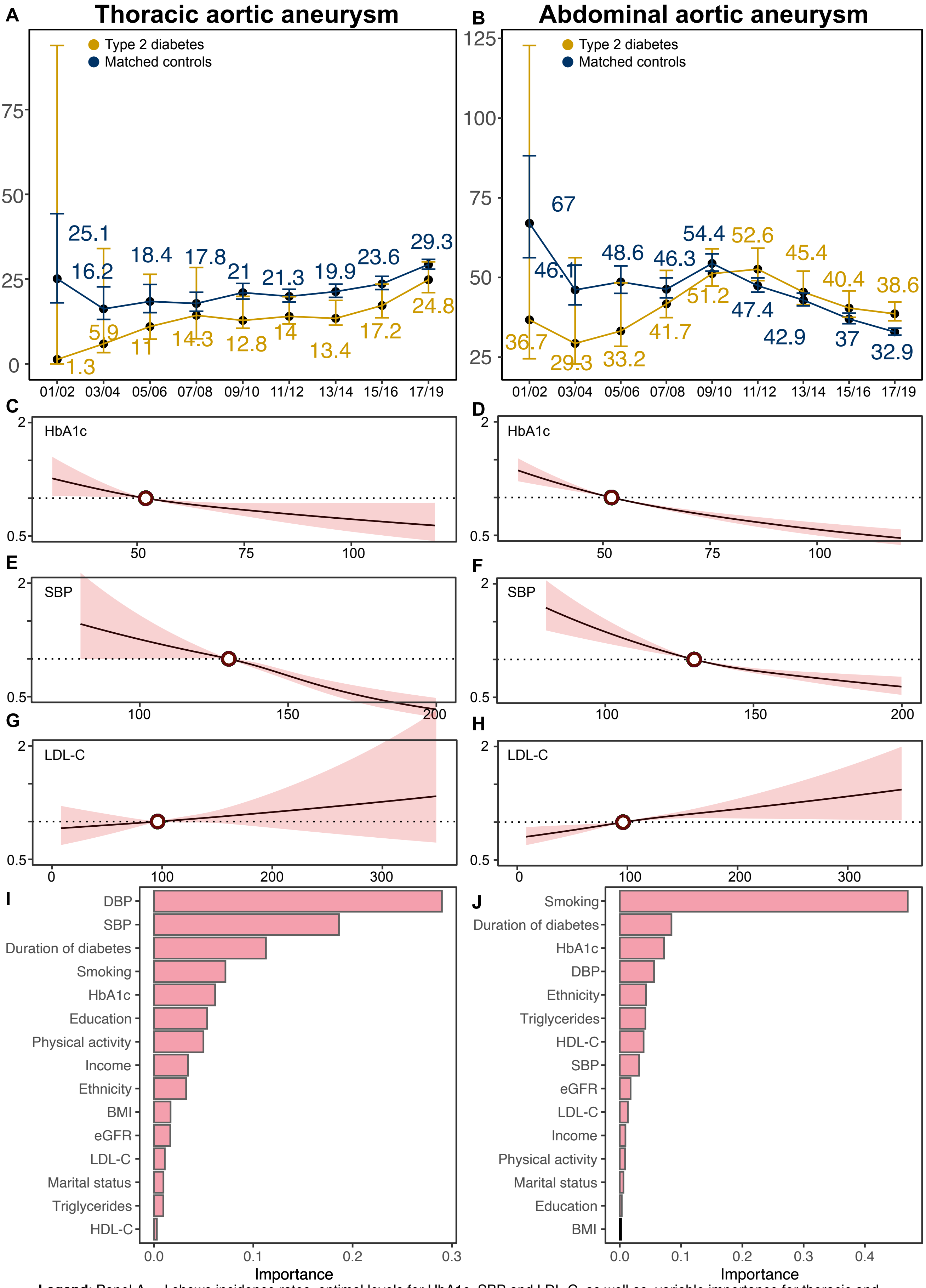

Legend: Panel A – J shows incidence rates, optimal levels for HbA1c, SBP and LDL-C, as well as, variable importance for thoracic and abdominal aortic aneurysm. Panel C-J shows results for patients with type 2 diabetes only.
